# Requisite Omega-3 HUFA Biomarker Thresholds for Preventing Murine Lupus Flaring

**DOI:** 10.3389/fimmu.2020.01796

**Published:** 2020-08-21

**Authors:** Kathryn A. Wierenga, Rita S. Strakovsky, Abby D. Benninghoff, Lichchavi D. Rajasinghe, Adam L. Lock, Jack R. Harkema, James J. Pestka

**Affiliations:** ^1^Department of Biochemistry and Molecular Biology, Michigan State University, East Lansing, MI, United States; ^2^Institute for Integrative Toxicology, Michigan State University, East Lansing, MI, United States; ^3^Department of Food Science and Human Nutrition, Michigan State University, East Lansing, MI, United States; ^4^Department of Animal, Dairy and Veterinary Sciences and USTAR Applied Nutrition Research, Utah State University, Logan, UT, United States; ^5^Department of Animal Science, Michigan State University, East Lansing, MI, United States; ^6^Department of Pathobiology and Diagnostic Investigation, Michigan State University, East Lansing, MI, United States; ^7^Department of Microbiology and Molecular Genetics, Michigan State University, East Lansing, MI, United States

**Keywords:** systemic lupus erythematosus, NZBWF1, ω-3 fatty acid, Omega-3 Index, highly unsaturated fatty acid, silica, docosahexaenoic acid, precision nutrition

## Abstract

Lupus is a systemic autoimmune disease typified by uncontrolled inflammation, disruption of immune tolerance, and intermittent flaring – events triggerable by environmental factors. Preclinical and clinical studies reveal that consumption of the marine ω-3 highly unsaturated fatty acids (HUFAs) eicosapentaenoic acid (EPA) and docosahexaenoic acid (DHA) might be used as a precision nutrition intervention to lessen lupus symptoms. The anti-inflammatory and pro-resolving effects of ω-3 HUFAs are inextricably linked to their presence in membrane phospholipids. The ω-3 HUFA score, calculated as [100 × (ω-3 HUFAs/(ω-3 HUFAs + ω-6 HUFAs))] in red blood cells (RBCs), and the Omega-3 Index (O3I), calculated as [100 × ((DHA+EPA)/total fatty acids)] in RBCs, are two biomarkers potentially amenable to relating tissue HUFA balance to clinical outcomes in individuals with lupus. Using data from three prior preclinical DHA supplementation studies, we tested the hypothesis that the ω-3 HUFA score and the O3I inversely correlate with indicators of autoimmune pathogenesis in the cSiO_2_-triggered lupus flaring model. The three studies employed both low and high fat rodent diets, as well as more complex diets emulating the U.S. dietary pattern. The ω-3 HUFA scores in RBCs were comparatively more robust than the O3I at predicting HUFA balances in the kidney, liver, spleen, and lung. Importantly, increases in both the ω-3 HUFA score (>40%) and the O3I (>10%) were strongly associated with suppression of cSiO_2_-triggered (1) expression of interferon-regulated genes, proinflammatory cytokine production, leukocyte infiltration, and ectopic lymphoid structure development in the lung, (2) pulmonary and systemic autoantibody production, and (3) glomerulonephritis. Collectively, these findings identify achievable ω-3 HUFA scores and O3I thresholds that could be targeted in future human intervention studies querying how ω-3 HUFA consumption influences lupus and other autoimmune diseases.

## Introduction

Systemic lupus erythematosus (lupus) is a prototypic, multifaceted autoimmune disease characterized by uncontrolled inflammation, disruption of self-tolerance, and intermittent episodes of disease flaring often triggered by environmental factors (1). Lupus-associated autoimmune pathogenesis elicits irreversible damage in the kidney and other organs, sometimes culminating in death. The overactive immune response in lupus is typically managed with glucocorticoids, which have deleterious effects associated with long-term use, including organ damage, osteoporosis, diabetes, and increased risk of cardiovascular disease (2, 3). Both animal and human studies indicate that consumption of marine ω-3 highly unsaturated fatty acids (HUFAs) docosahexaenoic acid (DHA) and eicosapentaenoic acid (EPA) may potentially alleviate the severity of chronic inflammatory and autoimmune diseases [reviewed in (4–6)], suggesting this precision nutrition approach might be a steroid-sparing intervention for lupus.

Human studies support the contention that ω-3 HUFA consumption may benefit lupus patients. In observational studies, low ω-3 HUFA intake is associated with exacerbated disease activity, adverse serum lipids, and atherosclerotic plaques in lupus patients (7), and a recent study by the Michigan Lupus Epidemiology and Surveillance (MiLES) program reported that positive patient-reported outcomes were associated with high consumption of ω-3 fatty acids and low dietary ω-6:ω-3 ratios (8). Most intervention trials implementing ω-3 HUFA supplementation in lupus patients report lessening of symptoms (9–18). However, there is variability across studies with some trials failing to show positive results. Key limiting factors contributing to disparities among investigations in humans include inadequate patient numbers; lack of consideration of effects of concurrent pharmacotherapies; variability in ω-3 HUFA dosages, sources, and supplementation durations; and failure to monitor ω-3 HUFA tissue levels in patients. This final point is immensely critical because the pro-resolving and anti-inflammatory properties of dietary ω-3 HUFAs are inextricably linked to the extent of their presence in the cell membrane (19). Importantly, pro-inflammatory ω-6 HUFAs, generated by elongation of shorter chain ω-6 polyunsaturated fatty acids (PUFAs), that dominate the typical Western diet compete with ω-3 HUFAs for occupancy at the sn2 position of phospholipids, thereby diminishing their anti-inflammatory and pro-resolving effects (19). In clinical studies, many factors influence the efficiency ω-3 HUFA incorporation, including patient compliance, individual differences in absorption, genetic variation in lipid metabolizing genes, and consumption of competing ω-6 PUFAs (20). Accordingly, for any clinical trial of marine ω-3 HUFA supplementation, it is essential to measure the balance of ω-3 HUFA levels both at baseline and throughout the study.

Animal models of lupus are an essential tool for understanding how gene-environment interactions influence development of the disease in humans. The NZBWF1 mouse is genetically predisposed to the development of autoimmune disease and has been widely used for over five decades as a preclinical lupus model for investigating mechanisms of disease pathogenesis, effects of environmental exposures, and efficacy of pharmacological and immunotherapeutic interventions (21). Female NZBWF1 mice spontaneously develop lupus at around 7 months of age, much earlier than males, and rarely live past 12 months (22, 23), mimicking the sex bias observed in human lupus. Inclusion of marine ω-3 HUFAs in the diet delays lupus onset and extends survival in this strain (24–29). Our laboratory has recently developed a novel model for lupus flaring involving intranasal instillation of female NZBWF1 mice with crystalline silica (cSiO_2_). Frequent, high exposure to cSiO_2_ particles in occupations such as construction, mining, and farming is etiologically linked to multiple human autoimmune diseases, including lupus (30–33). In this model, autoimmune disease is triggered 3 months earlier than vehicle-treated controls, as reflected in the lung by pro-inflammatory and interferon-regulated gene (IRG) upregulation, mononuclear cell infiltration, ectopic lymphoid structure (ELS) neogenesis, and autoantibody production. In the kidney, we see concurrent induction of glomerulonephritis (34, 35). Importantly, dietary supplementation with the ω-3 HUFA docosahexaenoic acid (DHA) ameliorates cSiO_2_-triggered lupus flaring in female NZBWF1 mice (35–38), and this intervention is effective against the background of three unique diets (36–38).

The ω-3 HUFA score (39) and the Omega-3 Index (O3I) (40) are two interrelated red blood cell (RBC) biomarkers potentially applicable for associating tissue HUFA balance with disease outcomes in both preclinical and clinical studies. The ω-3 HUFA score reflects the total ω-3 HUFAs as a % of total HUFAs (ω-3, ω-6, and ω-9 HUFAs), while the O3I is the sum of DHA and EPA as a percent of total fatty acids. The goal of the present study was to test the hypothesis that the ω-3 HUFA score and the O3I inversely correlate with indicators of inflammation and autoimmune pathogenesis during cSiO_2_-triggered lupus flaring in NZBWF1 mice. Data used to test this hypothesis were drawn from three unique DHA supplementation studies recently published by our laboratory (36–38) that employed both purified mouse diets, as well as more complex diets reflecting Western eating patterns. Our findings indicate that increases in both the O3I and the ω-3 HUFA score were strongly associated with suppression of autoimmune pathogenesis in this preclinical mouse model of toxicant-triggered lupus flaring. Importantly, these preclinical results identify the ω-3 HUFA score and O3I thresholds potentially required for successful intervention against lupus and other autoimmune diseases.

## Materials and Methods

### Experimental Design

Data used for this study were collected from our previously published investigations based on three DHA feeding studies (35–38) (see Supplementary Data). Each study used female NZBWF1 mice obtained from Jackson Laboratories (Bar Harbor, ME). Female mice were used in these studies due to the sexual dimorphism observed in both human lupus and the NZBWF1 mouse model (21). Experimental protocols were designed and performed in accordance with National Institutes of Health guidelines and approved by the Institutional Animal Care and Use Committee at Michigan State University (AUF#01/15-021-00; AUF# PROTO201800113). Upon arrival, mice were randomly assigned to experimental groups and housed four per cage with access to food and water provided *ad libitum*. Animal facilities were maintained at constant temperature (21–24°C) and humidity (40–55%) with a 12 h light/dark cycle. One animal in Study 3 was euthanized for health concerns unrelated to cSiO_2_ exposure or lupus development (38).

Experimental diets contained specified amounts of DHA against unique dietary backgrounds as summarized in Table 1. Study 1 used a modified high fat American Institute of Nutrition-93G diet (HF-AIN-93G) containing 134 g fat/kg diet (30% kcal fat), formulated with corn oil (10 g/kg), soybean oil (64 g/kg), and high-oleic safflower oil (60 g/kg) (35, 36). High-oleic safflower oil was substituted with 10, 30, or 60 g/kg microalgal oil containing 40% (w/w) DHA (DHASCO, provided by Dr. Kevin Hadley, Martek Biosciences Corporation, Columbia, MD). The resulting experimental diets yielded 0.4, 1.2, or 2.4% (w/w) DHA, respectively. Analyses were only performed on animals fed diets containing 0, 0.4, and 1.2% (w/w) DHA because no additional protection was seen when comparing the 1.2% (w/w) DHA diet to the 2.4% (w/w) DHA diet. Furthermore, animals fed the 2.4% (w/w) DHA diet achieved an ω-3 HUFA score of ~90%, which is much higher than those achieved in the other studies and beyond levels observed in humans (41). Study 2 employed the AIN-93G diet containing 70 g fat/kg diet (17% kcal fat), composed of corn oil (10 g/kg) and high-oleic safflower oil (60 g/kg) (37). High-oleic safflower oil was replaced with 10 or 25 g/kg DHASCO to yield experimental diets containing 0.4 and 1% (w/w) DHA, respectively. Study 3 utilized a modified total Western diet (MTWD) and a MTWD with 40% less saturated fats and ω-6 HUFAs (MTWD ↓SF.ω-6) (38). Both MTWDs contained 164 g fat/kg diet (34.5% kcal fat), composed of soybean oil, anhydrous milk fats, olive oil, lard, beef tallow, corn oil, cholesterol, and high-oleic safflower oil. Olive oil was replaced by 30 g/kg DHASCO to achieve 1.2% (w/w) DHA.

**Table 1:** Composition of experimental diets.

**Basal diet**	**Experimental diets**									
	**Study 1**^****36****^			**Study 2**^****37****^			**Study 3**^****38****^			
	**HF AIN-93G**			**AIN-93G**			**MTWD**		**MTWD** **↓SF**.**ω-6**	
**DHA (en%)**	**0%**	**0.96%**	**2.40%**	**0%**	**0.87%**	**2.62%**	**0%**	**2.63%**	**0%**	**2.63%**
**DHA (%w/w)**	**0%**	**0.40%**	**1.20%**	**0%**	**0.40%**	**1%**	**0%**	**1%**	**0%**	**1%**
**Macronutrient**	***(g/Kg)***									
**Carbohydrates**										
Corn starch	366	366	366	398	398	398	230	230	230	230
Maltodextrin (Dyetrose)	121	121	121	132	132	132	70	70	70	70
Sucrose	92	92	92	100	100	100	257	257	257	257
Cellulose	46	46	46	50	50	50	30	30	30	30
kcal (% of total)	53.3	53.3	53.3	63.2	63.2	63.2	49.4	49.4	49.4	49.4
**Proteins**										
Casein	184	185	184	200	200	200	190	190	190	190
L-cysteine	3	3	3	3	3	3	3	3	3	3
kcal (% of total)	16.7	16.7	16.7	19.7	19.7	19.7	16.1	16.1	16.1	16.1
**Fats**										
Soybean oil	64	64	64	-	-	-	29	29	6	6
Anhydrous milkfat	-	-	-	-	-	-	36	36	7	7
Olive oil^a, b^	-	-	-	-	-	-	30	0	138	108
Lard	-	-	-	-	-	-	28	28	6	6
Beef tallow	-	-	-	-	-	-	25	25	5	5
Corn oil^a, c^	10	10	10	10	10	10	16	16	3	3
Cholesterol	-	-	-	-	-	-	0.4	0.4	0.5	0.5
High-oleic safflower oil^a, d^	60	50	30	60	50	35	-	-	-	-
DHA-enriched algal oil^a, e^	0	10	30	0	10	25	0	30	0	30
kcal (% of total)	30.0	30.0	30.0	17.1	17.1	17.1	34.5	34.5	34.5	34.5
**Other**										
AIN93G mineral mix	32	32	32	35	35	35	41	41	41	41
AIN93G vitamin mix	19	19	19	10	10	10	12	12	12	12
Choline bitartrate	2	2	2	3	3	3	3	3	3	3
TBHQ antioxidant	0.01	0.01	0.01	0.01	0.01	0.01	0.03	0.03	0.03	0.03

a *Based on oil composition reported by manufacturer*.

b *Olive oil contained 678 g/kg oleic acid and 84 g/kg linoleic acid, as reported by the USDA, FDC ID 748648*.

c *Corn oil contained 612 g/kg linoleic acid and 26 g/kg oleic acid*.

d *High-oleic safflower oil contained 750 g/kg oleic acid and 140 g/kg linoleic acid*.

e *Algal oil contained 395 g/kg DHA and 215 g/kg oleic acid, as reported by manufacturer*.

In each study, groups of female mice (*n* = 7–8/group) were initiated on experimental diets at age 6 wk and maintained on those same diets until experiment termination. To limit oxidation of dietary lipids, diets were prepared every 2 weeks, vacuum-sealed and stored at −20°C, and provided fresh every 1–2 days. Two weeks later (age 8 wk), mice were anesthetized with 4% isoflurane and intranasally instilled with 1 mg cSiO_2_ (Min-U-Sil-5, 1.5–2.0 μm average particle size, U.S. Silica, Berkeley Springs, WV) in 25 μL PBS or PBS vehicle (VEH) every week for 4 weeks. The total amount of cSiO_2_ provided over the course of the experiment (4 mg per mouse) was chosen to approximate half of a recommended human lifetime exposure as established by the Occupational Safety and Health Administration (34). Mice were euthanized by intraperitoneally injecting 56 mg/kg BW sodium pentobarbital 11–13 weeks after the final cSiO_2_ exposure. Selected tissue analyses were conducted as described for Study 1 (36), Study 2 (35, 37), and Study 3 (38). These included fatty acid profiling (RBC, lung, kidney, spleen, liver), IRG expression (lung), pro-inflammatory cytokines (bronchioalveolar lavage fluid [BALF]), lymphocyte infiltration (lung), ELS development (lung), pulmonary and systemic autoantibody expression (BALF, plasma), and glomerulonephritis (kidney).

### Fatty Acid Analyses

Experimental diets from Studies 1, 2, and 3, tissues from Studies 1 and 3, and RBCs from Study 1 were analyzed by GLC at Michigan State University as described previously using a GC2010 Gas Chromatograph (Shimadzu, Columbia, MD) equipped with a CP-Sil 88 WCOT (wall-coated open tubular) fused-silica column (100 m × 0.25 mm i.d. × 0.2-μm film thickness; Varian Inc., Lake Forest, CA) with hydrogen as carrier gas (36). A standard cocktail of fatty acids characteristic of erythrocytes was used to identify phospholipid fatty acids, which were quantified as a percentage of total identified fatty acids after response factor correction. Analysis of RBCs from Studies 2 and 3 was performed by OmegaQuant Analytics, LLC (Sioux Falls, SD), an independent CLIA-certified laboratory.

To verify fatty acid compositions, final diets were analyzed by gas liquid chromatography (GLC) as described above and presented in Table 2. The dietary fatty acid composition was used to calculate predicted RBC ω−3 HUFA scores. We used a modification of Lands' equation (41) as follows, where HC3 = 3.0, HC6 = 0.70, PC3 = 0.0555, PC6 = 0.0441, HI3 = 0.005, CO = 5.0, and Ks = 0.175.

(1) $$\begin{array}{l} Predicted\;\omega -3\;HUFA\;Score=\\ 100-(\frac{100}{1+\left(HC6/en\%H6\right)\left(1+en\%H3/HC3\right)}+\\ \frac{100}{1+\left(PC6/en\%H6\right)\left(1+en\%P3/PC3+en\%H3/HI3+\;en\%O/CO+en\%P6/KS\right)}) \end{array}$$

**Table 2:** Fatty acid profiles of experimental diets as determined by gas-liquid chromatography.

	**Basal diet**	**Experimental diets**									
		**Study 1**^**36**^			**Study 2**^**37**^			**Study 3**^**38**^			
		**HF AIN-93G**			**AIN-93G**			**MTWD**		**MTWD** **↓SF**.**ω-6**	
	**DHA (en%)**	**0%**	**0.96%**	**2.40%**	**0%**	**0.87%**	**2.62%**	**0%**	**2.63%**	**0%**	**2.63%**
	**DHA (%w/w)**	**0%**	**0.40%**	**1.20%**	**0%**	**0.40%**	**1%**	**0%**	**1%**	**0%**	**1%**
**Common name**	**Chemical formula**	***(% of total fatty acids)***									
Lauric	C12:0	0.07 ± 0.00	0.30 ± 0.06	0.75 ± 0.03	0.04 ± 0.00	0.62 ± 0.00	1.42 ± 0.07	0.65 ± 0.01	1.49 ± 0.03	0.14 ± 0.01	0.83 ± 0.01
Myristic	C14:0	0.18 ± 0.01	0.97 ± 0.21	2.57 ± 0.11	0.16 ± 0.01	1.79 ± 0.02	4.15 ± 0.21	2.87 ± 0.06	5.54 ± 0.05	0.53 ± 0.01	2.51 ± 0.02
Pentadecanoic	C15:0	0.03 ± 0.00	0.03 ± 0.00	0.03 ± 0.00	0.03 ± 0.00	0.02 ± 0.00	0.03 ± 0.00	0.29 ± 0.00	0.30 ± 0.00	0.07 ± 0.00	0.06 ± 0.00
Palmitic	C16:0	8.31 ± 0.12	8.94 ± 0.33	10.05 ± 0.11	5.64 ± 0.04	6.82 ± 0.02	7.79 ± 0.02	22.24 ± 0.16	22.24 ± 0.05	10.87 ± 0.06	11.00 ± 0.01
Palmitoleic	C16:1ω7	0.09 ± 0.00	0.18 ± 0.02	0.34 ± 0.01	0.08 ± 0.00	0.41 ± 0.00	0.86 ± 0.03	0.13 ± 0.01	0.10 ± 0.00	0.13 ± 0.01	0.12 ± 0.01
Strearic	C18:0	2.61 ± 0.04	2.58 ± 0.07	2.47 ± 0.03	0.03 ± 0.00	0.04 ± 0.00	0.04 ± 0.00	8.49± 0.04	8.26 ± 0.03	3.61 ± 0.03	3.37 ± 0.01
Oleic	C18:1ω9	46.36 ± 0.87	42.55 ± 2.21	35.56 ± 1.05	71.08 ± 0.13	62.40 ± 0.08	53.26 ± 0.50	39.19 ± 0.38	29.00 ± 0.08	72.63 ± 0.08	64.41 ± 0.03
Linoleic	C18:2ω6	36.55 ± 0.70	36.09 ± 1.14	34.51 ± 0.70	19.17 ± 0.10	18.94 ± 0.01	15.08 ± 0.79	21.07 ± 0.21	20.62 ± 0.18	8.57 ± 0.04	7.82 ± 0.04
Arachidic	C20:0	0.32 ± 0.01	0.31 ± 0.01	0.28 ± 0.00	0.31 ± 0.00	0.28 ± 0.00	0.24 ± 0.00	0.21 ± 0.03	0.17 ± 0.01	0.29 ± 0.00	0.28 ± 0.01
Alpha-linolenic	C18:3ω3	3.15 ± 0.08	3.25 ± 0.08	3.26 ± 0.09	0.36 ± 0.01	0.37 ± 0.00	0.32 ± 0.01	1.90 ± 0.03	1.81 ± 0.04	0.93 ± 0.01	0.87 ± 0.02
Behenic	C22:0	0.28 ± 0.01	0.28 ± 0.01	0.28 ± 0.01	0.22 ± 0.00	0.21 ± 0.00	0.20 ± 0.00	0.10 ± 0.01	0.11 ± 0.01	0.10 ± 0.01	0.11 ± 0.00
Lignoceric	C24:0	0.13 ± 0.01	0.12 ± 0.01	0.12 ± 0.00	0.14 ± 0.00	0.13 ± 0.00	0.12 ± 0.00	0.04 ± 0.01	0.05 ± 0.01	0.03 ± 0.00	0.05 ± 0.00
Eicosapentaenoic	C20:5ω3	0.00 ± 0.00	0.00 ± 0.00	0.01 ± 0.00	0.00 ± 0.00	0.09 ± 0.00	0.20 ± 0.01	0.00 ± 0.00	0.13 ± 0.00	0.00 ± 0.00	0.10 ± 0.01
Docosahexaenoic	C22:6ω3	0.00 ± 0.00	2.47 ± 0.74	7.74 ± 0.43	0.00 ± 0.00	5.40 ± 0.05	14.20 ± 0.65	0.00 ± 0.00	7.09 ± 0.20	0.00 ± 0.00	6.34 ± 0.08
Total saturated fat		12.1 ± 0.2	13.7 ± 0.7	16.8 ± 0.2	8.3 ± 0.1	11.5 ± 0.1	15.4 ± 0.1	35.28 ± 0.21	38.84 ± 0.05	15.80 ± 0.08	18.39 ± 0.02
Total MUFA		47.8 ± 0.9	44.0 ± 2.2	37.1 ± 1.1	71.9 ± 0.1	63.5 ± 0.1	54.7 ± 0.5	41.74 ± 0.36	31.49 ± 0.10	74.70 ± 0.10	66.49 ± 0.01
Total ω-6 PUFA		36.7 ± 0.7	36.1 ± 1.14	34.6 ± 0.7	19.2 ± 0.1	18.9 ± 0.1	15.1 ± 0.8	21.07 ± 0.21	20.62 ± 0.18	8.57 ± 0.04	7.82 ± 0.04
Total ω-3 PUFA		3.2 ± 0.1	5.8 ± 0.9	11.1 ± 0.4	0.4 ± 0.00	5.9 ± 0.1	14.7 ± 0.1	1.90 ± 0.03	9.04 ± 0.19	0.93 ± 0.01	7.31 ± 0.05
ω-6:ω-3 ratio		11.6 ± 0.3	6.4 ± 0.9	3.12 ± 0.13	52.8 ± 1.3	3.2 ± 0.0	1.0 ± 0.1	11.07 ± 0.10	2.28 ± 0.07	9.24 ± 0.13	1.12 ± 0.01

*MUFA, monounsaturated fatty acid; PUFA, polyunsaturated fatty acid*.

En%P6 was the en% of linoleic acid (C18:2n6), en%P3 was the en% of alpha-linolenic acid, en%H6 was the en% of arachidonic acid (C20:4n6), and en%H3 was the en% of EPA (C20:5n3), DPA (C22:5n6), and DHA (C22:6n3). En%O (other fatty acids) was calculated for each diet by subtracting en%P6, P3, H6, and H3 from the total en% of fat in the diet. In diet formulations with no measurable arachidonic acid, EPA, DPA, or DHA, the values for en%H6 or H3 were replaced with 0.001, a value much smaller than the estimated en%H6 or H3 in the Western diet.

### Determination of the RBC ω-3 HUFA Score and the O3I

The ω-3 HUFA score and O3I were determined for RBCs and all available tissues of each animal. The ω-3 HUFA score is the sum of EPA (C20:5n3), DPA (C22:5n3), and DHA (C22:6n3) as a percentage of the most abundant HUFAs (C20:5n3, C22:5n3, C22:6n3, C20:3n6, C20:4n6, C22:4n6, C22:5n6, C20:3n9) (39).

(2) $$\begin{array}{l} \omega -3\;HUFA\;Score=\;\frac{100\%\;*\left(20:5n3\;+\;22:5n3\;+\;22:6n3\right)}{Total\;HUFA\;} \end{array}$$

The O3I was calculated by taking the sum of EPA and DHA as a percent of total fatty acids (40). In tissues, this value is referred to as EPA + DHA.

(3) $$\begin{array}{l} Omega-3\;Index\;\left(O3I\right)=\frac{100\%{\;}^{*}\left(20:5n3\;+\;22:6n3\right)}{Total\;FA\;} \end{array}$$

### Data Analysis and Statistics

All correlations to inflammatory endpoints used ω-3 HUFA scores and O3Is measured in RBCs. Data were analyzed using Graph Pad Prism 8.0.0 (GraphPad Software, San Diego, CA, www.graphpad.com). Inflammatory endpoints that were undetectable were replaced with half of the minimum value for the individual endpoint. The robust regression and outlier removal (ROUT) method was used to identified outliers, which were excluded from further analysis (*Q* = 0.5%). For all endpoints, <10% of data points were identified as outliers. Where appropriate, non-normal data were log_10_ transformed and analyzed using linear regression. To account for experimental and methodological differences, all log transformed inflammatory/autoimmune endpoints were standardized prior to performing correlations across multiple experiments. When the best-fit values of the slope and y-intercept were not significantly different between experiments, raw data from each experiment were combined and re-analyzed to obtain a single linear regression model. Correlation analyses were performed on raw data using Spearman's Correlation due to non-normality of the data (per Shapiro-Wilk Test, *p* < 0.05). For correlations to autoantibody classes and subtypes, autoantibody groups were determined based on location and function of cognate autoantigens. Within a given group, signal intensities for individual autoantibodies were normalized and summed to obtain a group score for each animal, as described previously (42). The score was used to perform correlation analyses against the RBC ω-3 HUFA score. In analyses comparing diet groups, data are presented as mean ± SEM with *n* = 7–8 mice per group. To compare the O3I and ω-3 HUFA scores of animals positive or negative for nephritis, the non-parametric Mann-Whitney Rank Sum test was used. A *p* < 0.05 was considered statistically different for all study outcomes.

## Results

### DHA Supplementation Dose-Dependently Increases ω-3 HUFA Score Uniformly Across RBC and Tissues

**Figure 1:**
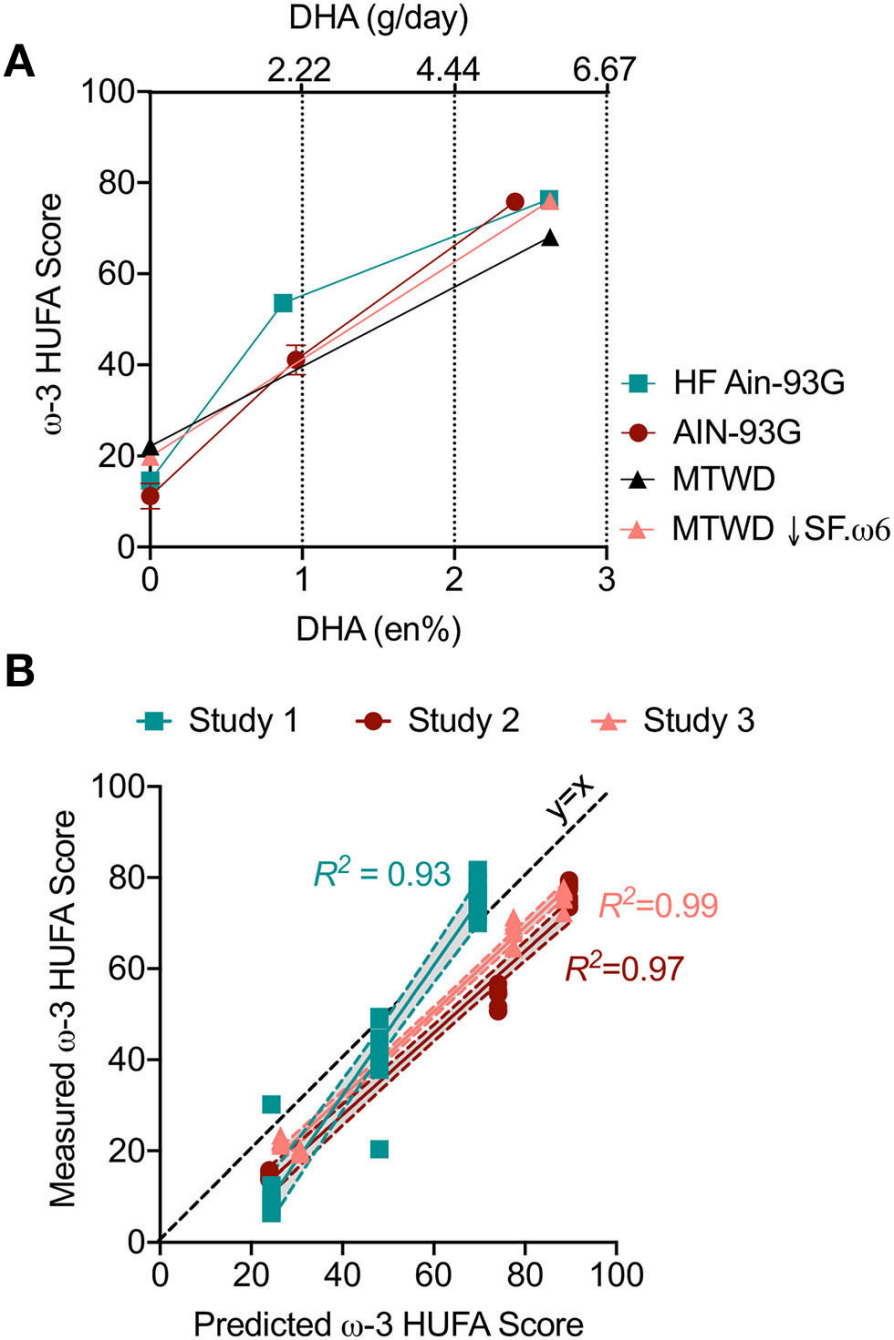
RBC ω-3 High unsaturated fatty acid (HUFA) score increases with DHA intake in NZBWF1 mice and can be predicted based on diet composition in cSiO_2_-treated NZBWF1 mice. Animals were fed different diets for Studies 1 (HF AIN-93G), 2 (AIN-93G), and 3 (MTWD and MTWD ↓SF.ω6) with or without DHA (see Table 1) as indicated by individually colored lines and symbols. At experiment termination, red blood cells (RBCs) were analyzed for fatty acids by GLC. **(A)** Increasing en% of DHA in the diet elevated omega-3 HUFA score similarly across all experimental diets. Data presented as mean ± SEM. **(B)** The ω-3 HUFA score is predictable based on the en% of major ω-3 and ω-6 fatty acids using Lands' equation. Individual animals represented by individual data points. For all regression analyses, *R*^2^ is reported next to the corresponding line and *p* < 0.001 Shaded bands around regression lines represent 95% confidence intervals.

**Figure 2:**
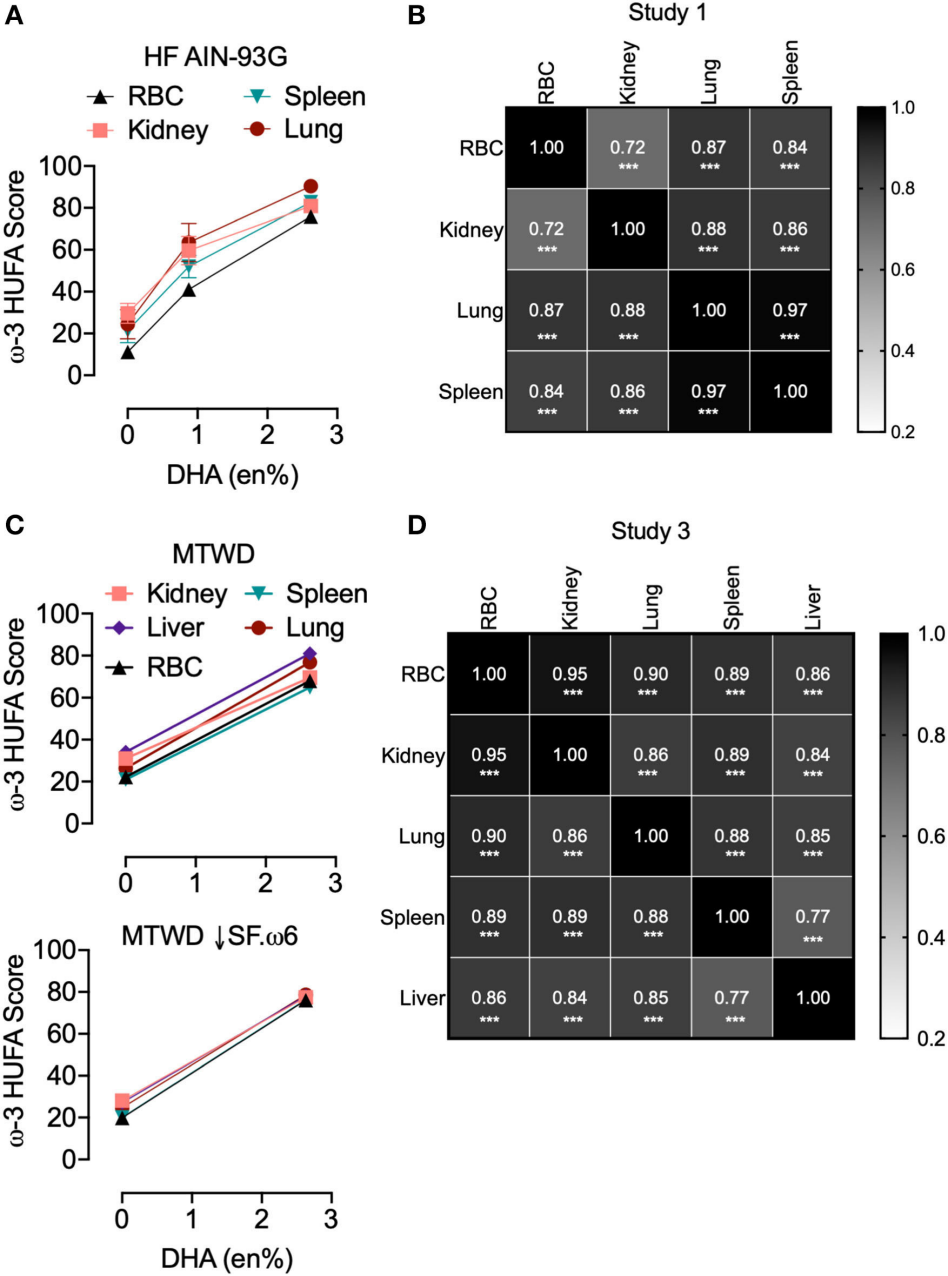
ω-3 HUFA scores are consistent across multiple tissues in cSiO_2_-treated NZBWF1 mice. Mouse tissues from **(A,B)** Study 1 (HF AIN-93G diet) and **(C,D)** Study 3 (MTWD and MTWD ↓SF.ω6 diets) were analyzed separately to assess the impact of DHA in tissue fatty acid incorporation. Study 2 is not included because only RBCs were analyzed in this study. **(A,C)** ω-3 HUFA scores increased similarly across tissues with DHA supplementation. Data presented as mean ± SEM. **(B,D)** Pearson's correlation was used to assess correlations between the ω-3 HUFA score across different tissues (***p < 0.001).

The effects of substituting various amounts of DHA-rich microalgal oil for high oleic acid safflower oil (HF AIN-93G, Study 1; AIN-93G, Study 2) or olive oil in (MTWD and MTWD ↓SF.ω6, Study 3) on resultant tissue and RBC ω-3 HUFA scores were compared. Regardless of diet, increasing the DHA content up to 2.6 en% (human equivalent dose of ~5 g/day) dose-dependently increased RBC ω-3 HUFA scores (Figure 1A). These increases closely correlated (*R*^2^ = 0.93–0.99, *p* < 0.05) with predicted ω-3 HUFA scores calculated from diet composition using Lands' equation (Figure 1B). ω-3 HUFA scores were relatively consistent across all tissues, both for basal and DHA-supplemented diets (Figures 2A,C). DHA-dependent increases in RBC ω-3 HUFA score closely correlated (*p* < 0.001) with those in lung (*r*_*s*_ = 0.87), spleen (*r*_*s*_ = 0.84), and kidney (*r*_*s*_ = 0.72) for Study 1 (Figures 2A,B), and in lung (*r*_*s*_ = 0.90), spleen (*r*_*s*_ = 0.89), liver (*r*_*s*_ = 0.86), and kidney (*r*_*s*_ = 0.95) for Study 3 (Figures 2C,D). Using the O3I as a measure of fatty acid content resulted in lower and more varied correlations (*r*_*s*_ = 0.27–0.88, Figures S2B,D).

### Elevated RBC ω-3 HUFA Scores Negatively Correlate With Interferon Regulated Gene (IRG) Expression in the Lung

**Figure 3:**
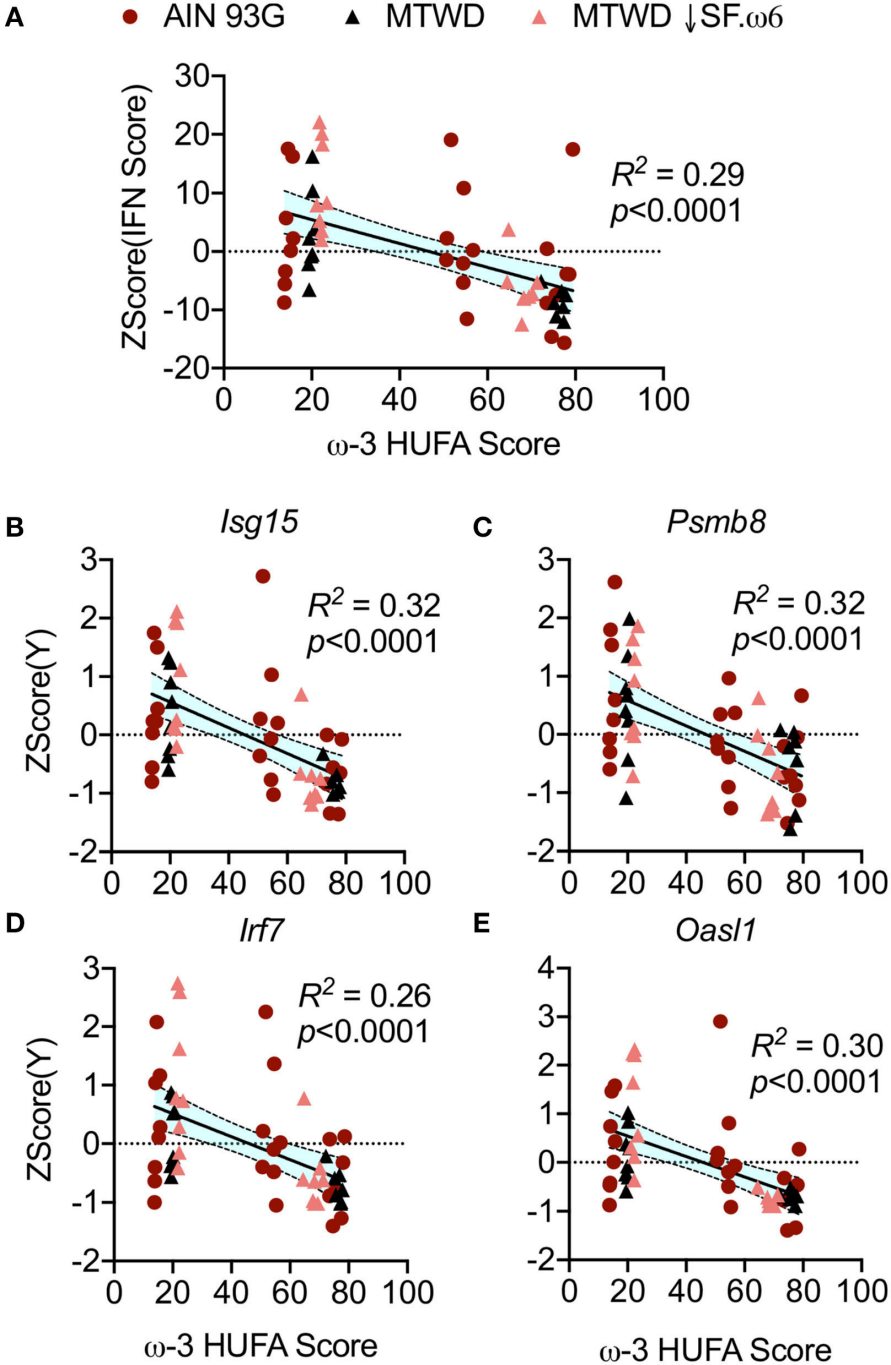
RBC ω-3 HUFA score negatively correlates with IFN regulated gene expression in cSiO_2_-triggered NZBWF1 mice. **(A)** An IFN score was calculated to include 12 IFN-related genes significantly induced by cSiO_2_ exposure (*Ccl7, Zbp1, Ifi44, Ifit1, Irf7, Isg15, Mx1, Oas2, Oasl1, IPsmb8, Rsad2, Siglec1*). These genes were presented as fold-change relative to vehicle-instilled animals. Missing values and outliers were handled as described in the Methods section. Expression was standardized by autoscaling (subtracting the mean expression of the gene and dividing by the standard deviation of the expression of the gene). Then, standardized scores of all genes for an individual sample were summed to achieve the IFN score **(B–E)**. Representative genes used in the calculation of the IFN score including **(B)** *Isg15*, **(C)** *Psmb8*, **(D)** *Irf7*, and **(E)** *Oasl1* reflect the trend observed in the combined IFN score. All values were plotted against the ω-3 HUFA score and the resulting data analyzed by simple linear regression. Regression coefficients were considered statistically significant at *p* < 0.05. Shaded bands around regression lines represent 95% confidence intervals.

Elevated IRG expression is highly associated with flaring and increased disease severity in lupus (43). It was demonstrated in Studies 2 and 3 that IRG expression is upregulated in cSiO_2_-exposed NZBWF1 mice and that this is suppressed by DHA supplementation (35, 38). Here, an IFN score was generated by combining the autoscaled expression of 12 IRGs measured in animals fed AIN-93G, MTWD, and MTWD ↓SF.ω6 (38). Resultant IFN scores negatively correlated with ω-3 HUFA scores (*R*^2^ = 0.29, *p* < 0.0001, Figure 3A). This negative correlation is illustrated for representative IRGs including *Isg15* (*R*^2^ = 0.32, *p* < 0.0001, Figure 3B), *Psmb8* (*R*^2^ = 0.32, *p* < 0.0001, Figure 3C), *Irf7* (*R*^2^ = 0.26, *p* < 0.0001, Figure 3D), and *Oasl1* (*R*^2^ = 0.30, *p* < 0.0001, Figure 3E). Overall, the autoscaled plots indicate that ω-3 HUFA scores above 40% were associated with reduced IRG scores and individual gene expression (Figure 3).

### Higher RBC ω-3 HUFA Scores Correspond to Reduced Pro-inflammatory Cytokines and Leukocyte Infiltration in BALF

**Figure 4:**
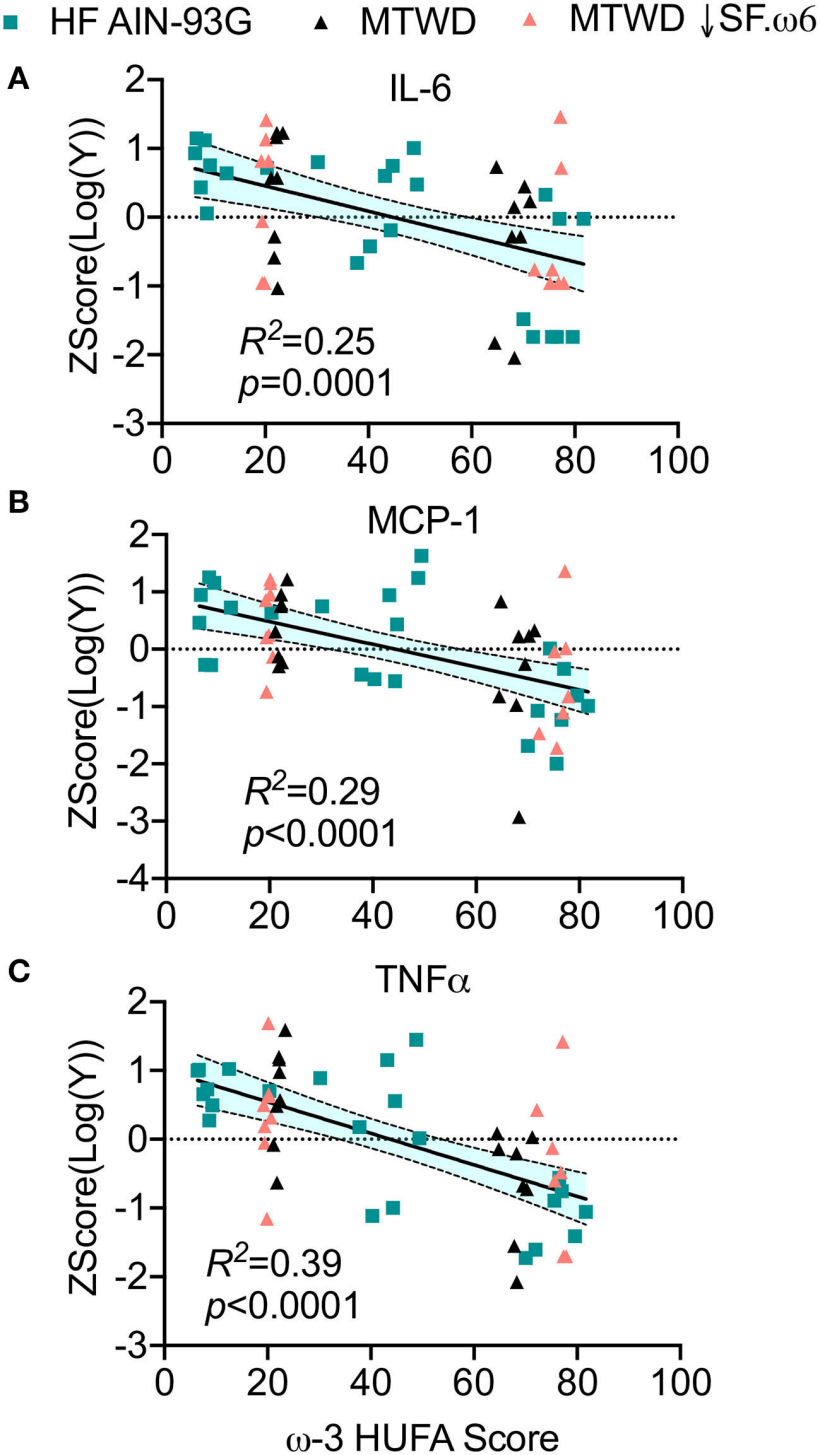
Increasing RBC ω-3 HUFA score corresponds to reduced inflammatory cytokines in the lung alveolar fluid of cSiO_2_-triggered NZBWF1 mice. Bronchoalveolar fluid (BALF) was analyzed for the proinflammatory cytokines **(A)** IL-6, **(B)** MCP-1, and **(C)** TNFα by ELISA in Study 1 and by a multiplexed bead based assay in Study 3. To compare across experiments, data was linearized by log_10_ transformation and standardized by autoscaling. The normalized and standardized data were plotted against the ω-3 HUFA score for each animal. When each diet was assessed individually, the resultant linear models were not found to be significantly different from one another, indicating that the data sets could be combined and analyzed simultaneously. The combined data were analyzed by a simple linear regression and goodness of fit presented as *R*^2^. Regression coefficients were considered statistically significant at *p* < 0.05. Shaded bands around regression lines represent 95% confidence intervals.

**Figure 5:**
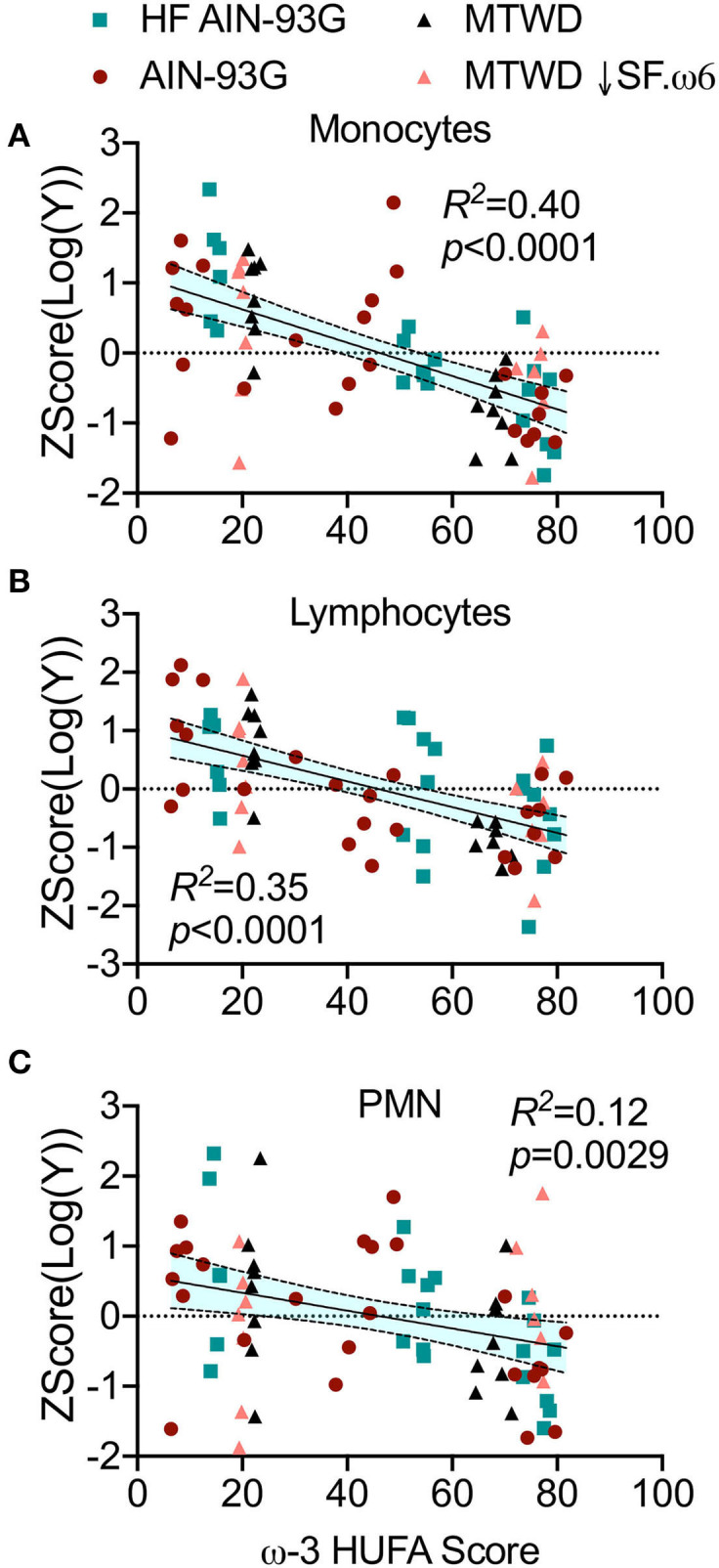
Elevated RBC ω-3 HUFA scores are associated with reduced mononuclear cell infiltration into lung alveolar fluid of cSiO_2_-triggered NZBWF1 mice. BALF was assessed for **(A)** macrophages, **(B)** lymphocytes, and **(C)** neutrophils by differential cell counts, as determined by morphological assessment of 200 total cells on cytological slides. Counts between diet groups were normalized by log transformation and standardized by autoscaling. The normalized and standardized data was plotted against the ω-3 HUFA score for each animal. The data was analyzed by a simple linear regression and goodness of fit presented as *R*^2^. Regression coefficients were considered statistically significant at *p* < 0.05. Shaded bands around regression lines represent 95% confidence intervals.

Intranasal instillation of cSiO_2_ elicits local sterile inflammation in the lungs of NZBWF1 mice that is associated with elevated proinflammatory cytokines, chemokines, and mononuclear cell influx, all of which can be suppressed by DHA supplementation (36–38). Here it was found that IL-6 (*R*^2^ = 0.26, *p* = 0.0001, Figure 4A), MCP-1 (*R*^2^ = 0.29, *p* < 0.0001, Figure 4B), and TNFα (*R*^2^ = 0.39, *p* < 0.0001, Figure 4C) concentrations in the BALF were negatively correlated with the ω-3 HUFA score. Consistent with these findings, numbers of macrophages (*R*^2^ = 0.40, *p* < 0.0001, Figure 5A), lymphocytes (*R*^2^ = 0.35, *p* < 0.0001, Figure 5B), and neutrophils (*R*^2^ = 0.12, *p* = 0.0029, Figure 5C) in BALF also negatively correlated with the ω-3 HUFA score in all three studies. Though some *R*^2^ values are relatively low, there is a consistent negative linear relationship with all endpoints assessed. Consonant with IRG expression, reductions in these inflammatory responses was most apparent when ω-3 HUFA scores exceeded 40% (Figures 4, 5).

### Increased RBC ω-3 HUFA Scores Are Associated With Reduced Ectopic Lymphoid Structure (ELS) Neogenesis and Autoantibody Production

**Figure 6:**
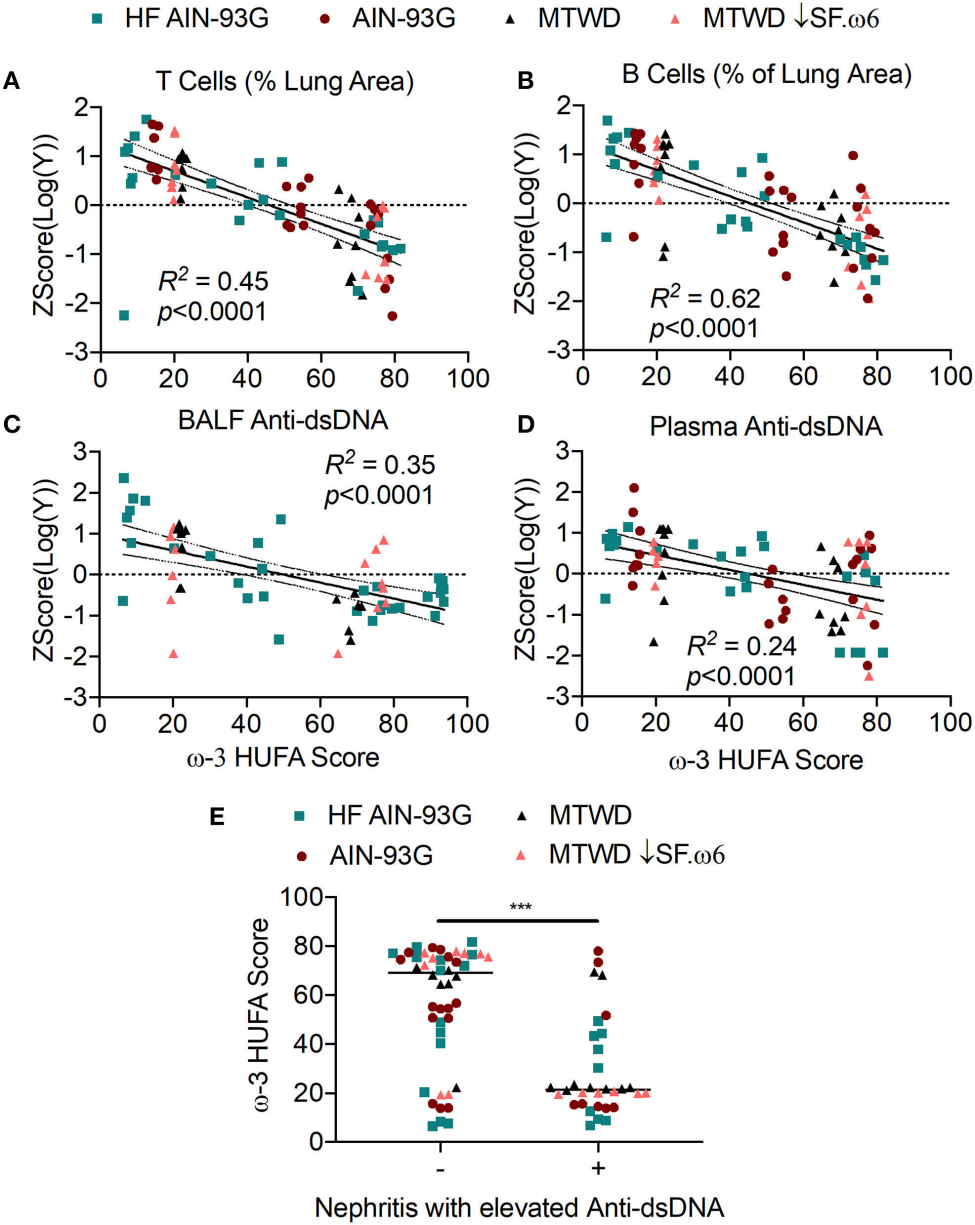
High RBC ω-3 HUFA scores correspond with suppression of ectopic lymphoid structure (ELS) neogenesis, anti-dsDNA response, and disease progression in cSiO_2_-triggered NZBWF1 mice. **(A,B)** ELS neogenesis was assessed by measuring the volume density of **(A)** T cells (CD3^+^) and **(B)** B cells (CD45R^+^), respectively, in the bronchial and perivascular regions of the lung. Anti-dsDNA was measured in **(C)** BALF and **(D)** plasma by ELISA. Percent area covered by T or B cells and anti-dsDNA levels and were log_10_ transformed to normalize followed by autoscaling to standardize across experiments. These values were plotted against the ω-3 HUFA score and the resulting data analyzed by simple linear regression. Goodness of fit of the linear regression was presented as *R*^2^. Regression coefficients were considered statistically significant at *p* < 0.05. Shaded bands around regression lines represent 95% confidence intervals. **(E)** Mice positive for renal lesions and elevated plasma anti-dsDNA IgG (significantly different from mean of the Veh-treated group, *p* < 0.05) had significantly lower median ω-3 HUFA scores than mice in the the group negative for these endpoints, as assessed by the non-parametric Mann-Whitney *U*-test (***p < 0.001). Quantification of renal histopathology score was based on the following scoring criteria: No proteinosis, normal glomeruli (0); multifocal segmental proliferative glomerulonephritis (1); multifocal segmental proliferative glomerulonephritis and occasional glomerular sclerosis and crescent formation (2); diffuse global segmental proliferative glomerulonephritis (3). Animals receiving any score ≥1 were categorized as positive for renal lesions.

**Figure 7:**
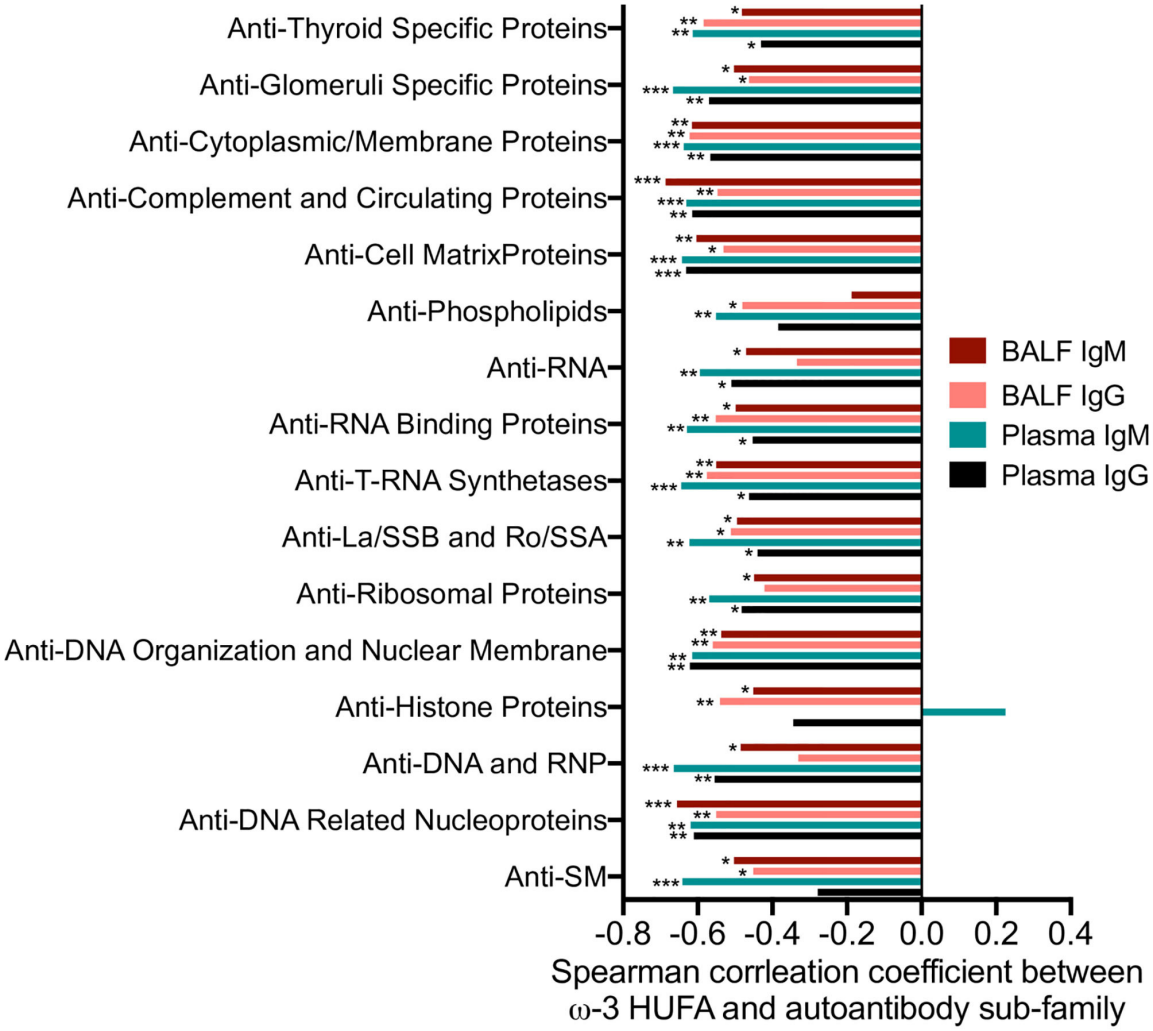
Increased RBC ω-3 HUFA scores correlate with reductions in a broad array of autoantibodies relative to specificity and isotype in the plasma and BALF of cSiO_2_-treated NZBWF1 mice. Autoantigen coated protein arrays were used for profiling four isotypes of autoantibody (IgG, IgM, IgA, and IgE) in plasma and BALF in Study 2. The final intensity value of each autoantibody was expressed as an autoantibody score. Individual autoantibodies were grouped according to the function of their cognate antigens (group names shown on y-axis) as described in the Methods section. The scores of each autoantibody in this group were combined to obtain an overall score for each group. This score was related to the ω-3 HUFA score using Spearman's correlation coefficient. *p < 0.05, **p < 0.01, ***p < 0.001.

Central to cSiO_2_-triggered autoimmunity in NZBWF1 mice is the appearance of ELS in the lung composed of germinal center-like organization of B- and T-cells (36). These structures promote the development of autoreactive plasma cells and the production of autoantibodies. Notably, their formation is suppressed by DHA supplementation (36–38). Consistent with those observations, very strong, negative linear correlations were observed between the ω-3 HUFA score and CD3^+^ (*R*^2^ = 0.45, *p* < 0.0001) and CD45R^+^ (*R*^2^ = 0.62, *p* < 0.0001) lung tissue in Studies 1, 2, and 3 (Figures 6A,B). Similar correlations were observed for anti-dsDNA in BALF (*R*^2^ = 0.35, *p* < 0.0001) and plasma (*R*^2^ = 0.24, *p* < 0.0001) as measured by ELISA (Figures 6C,D). Again, ω-3 HUFA scores over 40% were associated with reduced ELS development and anti-dsDNA production (Figure 6). A further feature of Study 2 was the use of high throughput autoantigen microarray for in-depth analysis of autoantibodies relative to specificity and isotype (44). Robust negative correlations were found between ω-3 HUFA score and IgG and IgM autoantibodies in both BALF and plasma with specificity for a broad range of host antigens (most *r*_*s*_ values between −0.4 and −0.6, significance indicated by asterisks) (Figure 7).

### Higher RBC ω-3 HUFA Scores Were Associated With Delayed Disease Progression

Early glomerulonephritis onset and production of autoantibodies is a critical outcome of cSiO_2_-triggered systemic autoimmunity that was prevented by dietary DHA supplementation in Studies 1, 2, and 3 (36–38). We defined lupus disease progression in animals as the presence of renal lesions combined with elevated plasma anti-dsDNA IgG in cSiO_2_-treated animals compared to the mean of the vehicle-treated group (*p* < 0.05). This is reflective of the SLICC criteria published in 2013, which stated that combination of biopsy confirmed nephritis in the presence of either ANA or anti-dsDNA antibodies is sufficient for classification of SLE in humans (45). Mice negative for both renal lesions and plasma anti-dsDNA IgG had significantly higher ω-3 HUFA scores (median of 69.18, 95% CI 54.46–74.59) compared to animals positive for both endpoints (median of 21.43, 95% CI 19.44–30.21) (Figure 6E). Consistent with the above findings for inflammation and autoimmunity indicators, ω-3 HUFA scores below ~40% were associated with disease progression.

### Higher O3I Were Associated With Reduced Autoimmune Pathogenesis

O3Is for Study 1 increased with en% DHA in the diet to a much lesser extent than those for Studies 2 and 3 (Figure S1). When assessing DHA's effects on the O3I in tissues, responses followed the rank order of kidney > lung > spleen > RBC for Study 1, whereas for Study 3 the rank order was RBC > kidney > lung > spleen > liver (Figures S2A,C). Previous reports of the RBC O3I for animals fed similar diets were much more similar to those observed in studies 2 and 3 (in the range of 6–14%) (46). Together these observations suggest that there were methodological issues with the fatty acid analysis in Study 1, possibly due to fatty acid decomposition. Therefore, correlation analyses between O3Is and inflammation and autoimmunity indicators were performed only for Studies 2 and 3.

**Figure 8:**
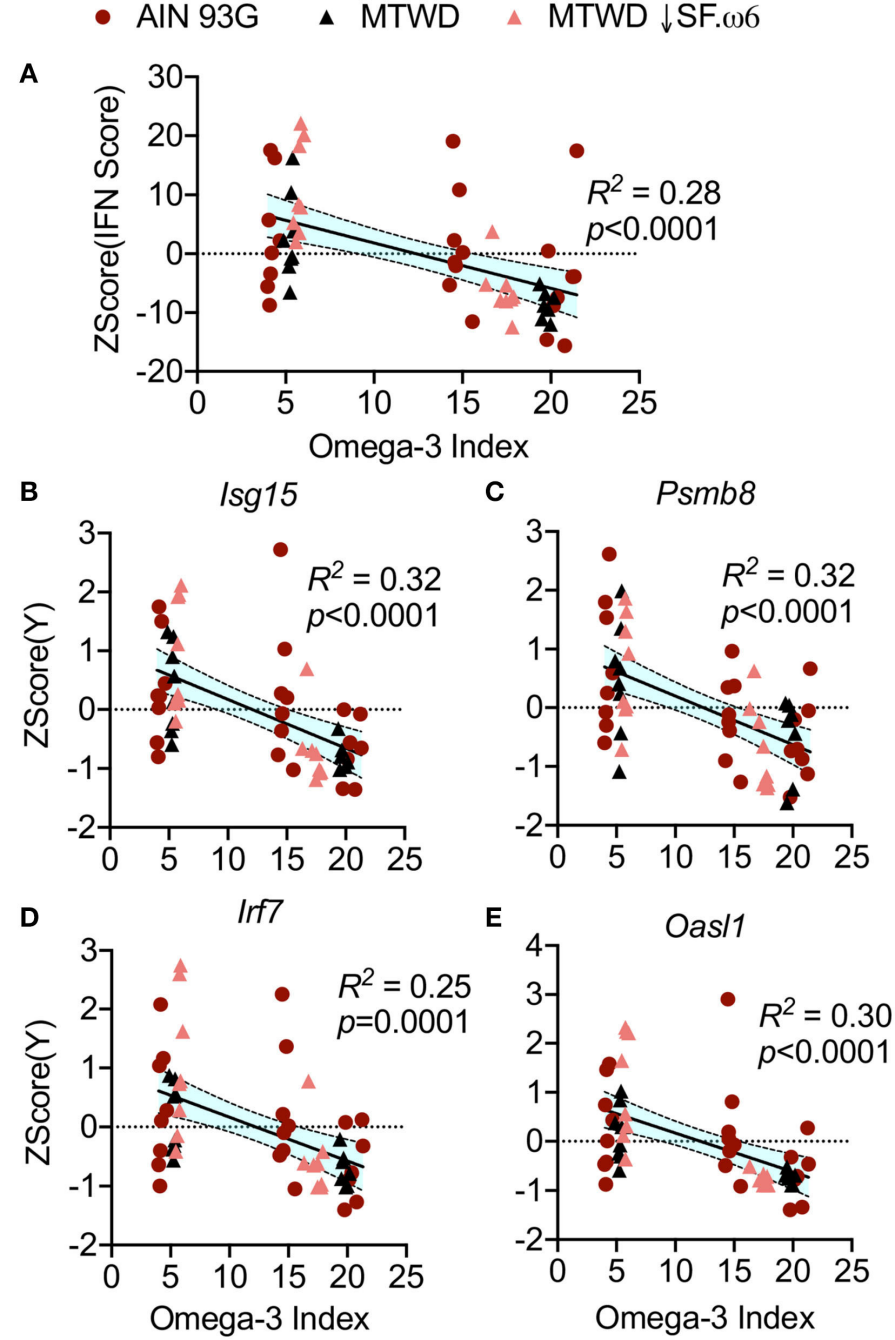
The Omega-3 Index (O3I) negatively correlates with IRG expression in cSiO_2_-triggered NZBWF1 mice. The IFN scores **(A)** and expression of **(B)** *Isg15*, **(C)** *Psmb8*, **(D)** *Irf7*, and **(E)** *Oasl1* expression were calculated as described in Figure 3. The autoscaled IFN scores and the expression of each gene was plotted against the O3I and the resulting data analyzed by simple linear regression. Regression coefficients were considered statistically significant at *p* < 0.05. Shaded bands around regression lines represent 95% confidence intervals.

**Figure 9:**
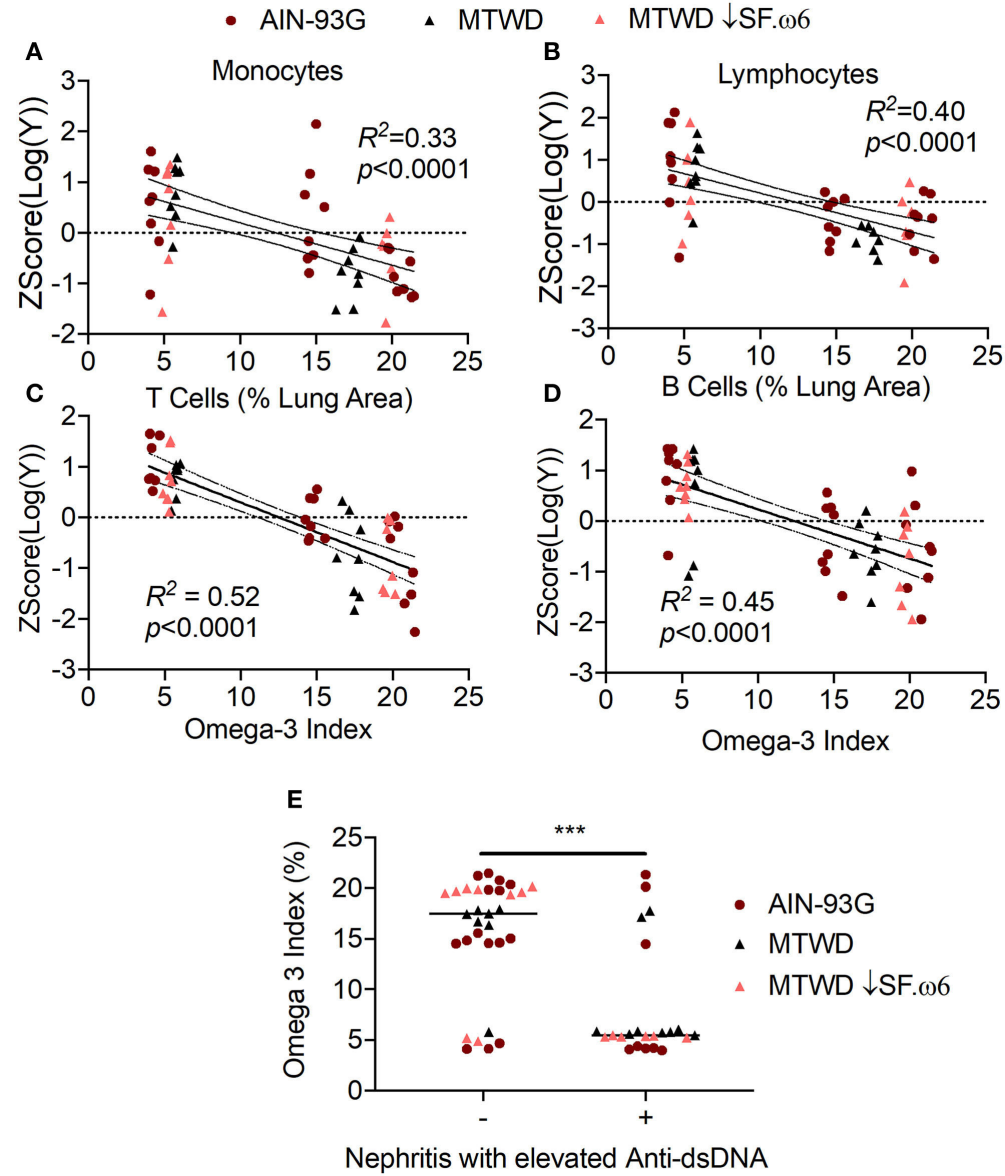
Heightened O3Is correspond with suppression of leukocyte infiltration, ELS development, and disease progression in cSiO_2_-triggered NZBWF1 mice. **(A)** Macrophage and **(B)** lymphocyte infiltration, as well as **(C)** B-cell, and **(D)** T-cell positive lung tissue were negatively correlated with the O3I. **(E)** Mice positive for renal lesions and elevated plasma anti-dsDNA IgG (significantly different from mean of the Veh-treated group, *p* < 0.05) had significantly lower median Omega-3 Indexes than mice in the the group negative for these endpoints, as assessed by the non-parametric Mann-Whitney *U*-test (***p < 0.001). For **(A–D)**, data were analyzed by a simple linear regression and goodness of fit presented as *R*^2^. Regression coefficients were considered statistically significant at *p* < 0.05. Shaded bands around regression lines represent 95% confidence intervals.

O3Is significantly correlated with decreased IFN scores (Figure 8A) and with downregulated expression of the representative IRGs *Isg15* (*R*^2^ = 0.28, *p* < 0.0001, Figure 8B), *Psmb8* (*R*^2^ = 0.32, *p* < 0.0001, Figure 8C), *Irf7* (*R*^2^ = 0.25, *p* = 0.0001, Figure 8D), and *Oasl1* (*R*^2^ = 0.30, *p* < 0.0001, Figure 8E). Furthermore, high O3Is were strongly associated with suppression of cSiO_2_-triggered increases in numbers of macrophages (*R*^2^ = 0.33, *p* < 0.0001, Figure 9A) and lymphocytes (*R*^2^ = 0.40, *p* < 0.0001, Figure 9B) in BALF, as well as decreased ELS neogenesis in the lung as reflected by B-cell (*R*^2^ = 0.52, *p* < 0.0001, Figure 9C) and T-cell (*R*^2^ = 0.45, *p* < 0.0001, Figure 9D) accumulation. Importantly, autoscaled plots consistently suggested that O3Is above 10% were associated with reduced IRG expression, leukocyte infiltration, and ELS development (Figures 8, 9). Lastly, O3Is were significantly lower in mice (median of 5.44, 95% CI 5.30–5.85) that showed development of lupus as indicated by renal lesions and elevated anti-dsDNA compared to mice negative for both of these endpoints (median of 17.48, 95% CI 14.83–19.67) (Figure 9E).

**Figure 10:**
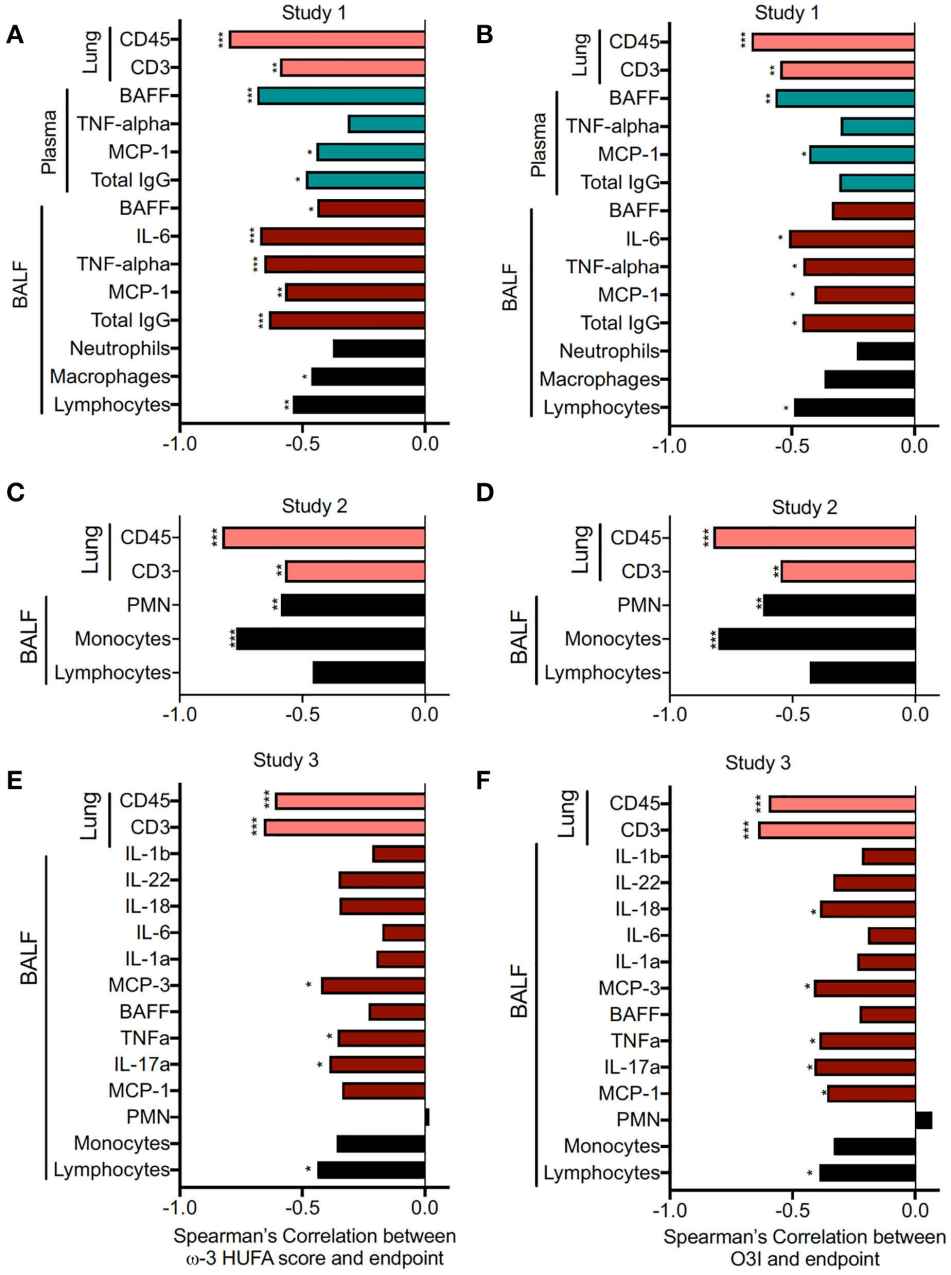
RBC ω-3 HUFA score and O3I both negatively correlate with inflammatory/autoimmune indicators and pulmonary immune cell infiltration. Correlation between inflammatory endpoints and RBC ω-3 HUFA scores and O3Is for individual animals was assessed by Spearman's correlation coefficient, due to non-normal distribution of samples. Many endpoints in Study 1 (HF AIN-93G diet) **(A,B)**, Study 2 (AIN-93G diet) **(C,D)**, and Study 3 (MTWD and MTWD ↓SF.ω6 diets) **(E,F)** were significantly negatively correlated with both the omega-3 HUFA score **(A,C,E)** and the O3I **(B,D,F)**. *p < 0.05, **p < 0.01, ***p < 0.001.

Correlations between inflammation/leukocyte infiltration indicators and RBC ω-3 HUFA scores and O3I for individual animals in Studies 1, 2, and 3 were assessed by Spearman's correlation analysis. Both ω-3 HUFA scores and the O3I were found to similarly negatively correlate with most endpoints in each study, suggesting that both biomarkers were comparable in predicting DHA's disease-preventive effects (Figure 10). The only endpoint that showed an opposing trend was the number of PMN measured in the BALF. These differences may be due to the fact that the animals in each experiment were sacrificed at slightly different times post cSiO_2_ instillation. It appears that animals sacrificed at later dates show an increasing strength in the correlation between ω-3 content and PMN (study 3 sacrificed at 11 weeks, study 2 sacrificed at 13 weeks, study 1 sacrificed at 12 weeks). This may be due to increased disease severity leading to more pronounced neutrophil infiltration between treatment groups.

## Discussion

Murine lupus models typically display gradual increases in autoantibodies prior to glomerulonephritis and thus mimic quiescent disease prior to flaring-associated organ damage (47). Here, airway exposure to cSiO_2_ was used to mimic flaring in NZBWF1 mice by promoting persistent sterile inflammation, cell death, robust expression of IRGs, and development of autoantibody-producing ELS in the lung (34–36). These autoantibodies and resultant immune complexes can accumulate in the kidney, accelerating glomerulonephritis (48–50). We report here for the first time that increasing two biomarkers of ω-3 HUFA tissue content, the ω-3 HUFA score and the O3I, by dietary DHA supplementation is highly associated with suppression of cSiO_2_-triggered lupus flaring. Benchmark thresholds for these biomarkers were further identified that may be highly relevant to future clinical use of ω-3 HUFA supplementation as an intervention against lupus and other autoimmune diseases.

As has been reviewed previously (51), autoimmune disease onset and progression following cSiO_2_ inhalation likely begins with unresolvable inflammation and rampant cell death in the lung, overwhelming the ability of alveolar macrophages to clear autoantigen-containing debris by efferocytosis (52). The presence of host nucleic acids released from dying cells may stimulate a type I IFN response (53, 54). Type I IFNs, including IFN-α, promote autoantigen presentation to infiltrating B- and T-cells and induce the release of additional cytokines such as B-cell activating factor (BAFF) (55, 56), the target of the monoclonal antibody drug Benlysta, approved for treatment of adult lupus in 2011 and pediatric lupus in 2019. BAFF stimulates the maturation of B-cells into autoantibody-producing plasma cells. The resultant DNA-containing immune complexes induce further release of IFN-α, sustaining this cycle (43). Marine ω-3s and their metabolites attenuate multiple steps of this putative pathway, resulting in protection against cSiO_2_-triggered autoimmunity. Several studies indicate that DHA is capable of blocking key inflammatory pathways and promoting a more pro-resolving phenotype in macrophages, which enhances their ability to efferocytose dying cells, thereby preventing aberrant production of type 1 IFNs, pro-inflammatory cytokines, and chemokines (57–59). Together, these inhibitory actions could dampen the subsequent inflammatory and downstream autoimmune responses. As shown here, increasing both the ω-3 HUFA score or the O3I correlated with reductions in IRG, cytokine, chemokine expression, B- and T-cell infiltration, autoantibody production, and glomerulonephritis induced by cSiO_2_ exposure.

In 2020, the National Institutes of Health announced at 10 years strategic plan focusing on precision nutrition—a “holistic approach to developing comprehensive and dynamic nutritional recommendations relevant to both individual and population health” (https://www.niddk.nih.gov/about-niddk/strategic-plans-reports/strategic-plan-nih-nutrition-research). Selection of dietary lipids would be central to the development on an individual's precision nutrition plan. The strong correlations between the ω-3 biomarkers and inflammatory endpoints suggest that the balance between ω-3 and ω-6 fatty HUFAs in the cell membrane is critical to promoting inflammation or resolution (60). At the translational level, there are a variety of factors that will influence the incorporation of dietary HUFAs into the cell membrane of individuals. ω-3 and ω-6 HUFAs compete for incorporation into the membrane phospholipids at the sn2 position, thus increasing the levels of ω-6 fatty acids in the diet will reduce the ω-3 HUFA incorporation in the tissue and vice versa (19). It has also been shown that the bioavailability of ω-3 supplements is enhanced when provided with a meal rich in other fats (61). Finally, single nucleotide polymorphisms (SNPs) in lipid metabolizing genes are associated with altered levels of various fatty acids observed in the RBCs and tissues (62, 63), and variations in lipid metabolizing genes are associated with the efficacy of ω-3 supplementation in cardiovascular disease (CVD) (64). Therefore, in preclinical and clinical ω-3 HUFA intervention studies, it is vital to measure of the balance of ω-3 and ω-6 HUFAs.

Measuring an individual's tissue HUFA status can be readily accomplished with low-cost commercial tests that are performed using dried blood spots (65). The alteration of RBC ω-3 and ω-6 fatty acids observed following dietary interventions is reflected in multiple tissues (Figure 2, Figure S2). Similarly, other studies have shown membrane fatty acid profiles of various immune cells, including monocytes, macrophages, T-cells, and B-cells, are also influenced by ω-3 supplementation (66). Of the two biomarkers studied, the O3I (i.e., DHA + EPA as a percent of total erythrocyte fatty acids) has been extensively validated in human clinical studies and is more widely implemented. A critical advantage of the O3I is the wealth of literature utilizing this biomarker, which was proposed for use as a risk factor for CVD in 2004. The widespread use of this biomarker has allowed for meta-analyses to identify O3I levels that show protection against a variety of disease endpoints, particularly in the field of cardiovascular and coronary heart disease. In instances where storage conditions, extraction protocols, and analytical techniques remain consistent across studies and samples, the O3I is preferable because it can be understood in the context of previous studies. If inconsistencies among these factors are a concern, defining fatty acid levels using the ω-3 HUFA score might be more advantageous.

A major advantage of the ω-3 HUFA score is its consistency across tissues and blood fractions and recalcitrance to differences in storage conditions and analytical techniques. Since HUFAs have similar chemical properties, they are degraded at similar rates (19). This appeared to be critical factor in our studies, where we found that ω-3 HUFA scores in RBCs were similarly impacted by en% DHA across all three studies, whereas the O3I was less robust (Figure S1). This observation is supported by a previous study that investigated the stability of dried blood spot fatty acids over the course of 4 weeks when stored at 4 and −20°C. Bell et al. observed a 15–30% decrease in individual HUFAs stored at 4°C for 28 days, while the ω-3 HUFA score decreased by only 8% (67). Because our samples were stored and processed under different conditions among experiments, we favored the use of the ω-3 HUFA score for this study. With the ω-3 HUFA score, we saw slightly higher correlations in many of the endpoints assessed, which may be in part due to the reduced variability observed in the HUFA score compared to the O3I. Another advantage shown here (Figure 1B) and previously (41) is that the ω-3 HUFA score can be predicted from dietary fat intake, making it an important tool when developing personalized nutritional interventions. Finally, focusing on the HUFA pool gives the clinician insight into the potential for the generation of anti-inflammatory ω-3 and proinflammatory ω-6 HUFA metabolites (19, 68).

There are multiple mechanisms by which ω-3 and ω-6 HUFAs directly influence inflammatory pathways in the cell (69). First, by increasing membrane fluidity and impeding lipid raft formation, DHA and EPA can interfere with activation of transmembrane receptors associated with inflammatory signaling (70). Second, both extracellular and intracellular phospholipases can cleave HUFAs from the membrane (71, 72). Resultant free DHA and EPA may activate transmembrane receptors or intracellular receptors associated with suppressing proinflammatory signaling (73, 74). Specifically, ω-3 HUFAs have been shown to antagonize TLR activation (75, 76) and interfere with NF-kB-dependent transcription by activating PPARγ (58, 77). Third, both DHA and EPA are metabolized to form specialized pro-resolving mediators (SPMs) such as maresins, resolvins, protectins, and anti-inflammatory epoxide metabolites (78, 79). SPMs inhibit inflammatory signaling (80, 81) and promote efferocytosis of dead cells (82, 83), both of which are critical to halting autoimmune disease pathogenesis.

Besides competing for cell membrane incorporation, ω-3 HUFAs can inhibit ω-6 HUFA metabolism to downstream proinflammatory eicosanoids (e.g., thromboxanes, prostaglandins, and leukotrienes) (19). Lipid metabolites derived from the arachidonic acid cascade have primarily inflammatory actions, especially during acute inflammation. Shifting the HUFA balance to favor ω-3 HUFAs rather than ω-6 HUFAs, such as arachidonic acid, may enhance the pro-resolving phenotype promoted by ω-3 derived lipid mediators. A recent study demonstrated that the plasma and red blood cell levels of ω-3 HUFAs were highly correlated with the production of downstream lipid mediators (79). Similarly, supplementation with EPA and DHA led to a decrease in ω-6 HUFAs, namely arachidonic acid, as well as decreased ω-6 HUFA-derived metabolites.

It is likely that the anti-inflammatory actions of ω-3 HUFAs and their downstream metabolites are at play in the inflammatory processes driving lupus symptoms. Among lupus patients, higher ω-3 HUFA levels or more frequent consumption of fish correlate with reduced disease activity (8, 84). In 2011, it was reported that lupus patients had lower amounts of ω-3 HUFAs in RBC and plasma than observed in healthy controls (85), and a subsequent study showed a negative correlation between adipose ω-3 levels and disease activity (7). More recently, it was shown that individuals with lupus had decreased levels of plasma resolvin D1, an anti-inflammatory metabolite of DHA, as compared to healthy controls (86). To date, there has been no extensive study of the membrane fatty acid content or plasma lipidome of lupus patients. Investigation in this area is necessary to elucidate potential benefit of ω-3 supplementation in human patients.

The majority of clinical trials utilizing ω-3 fatty acid supplementation to combat disease have been specific to CVD. Over the past three decades, randomized control trials (RCTs) have produced inconclusive results, with some showing benefit and others not. There are a variety of potential reasons for this inconsistency, as thoroughly reviewed by Rice et al. (87). Reasons include, among other things, insufficient dose of ω-3 HUFAs and inadequate duration of supplementation. Analysis of the results of some CVD studies reveal that that there can be significant overlap in the O3I in treatment vs. control group at trial completion, which would explain why researchers did not observe any effect with supplementation (88, 89). Additionally, there is a lack of consistency in measuring the fatty acid content in trial participants. The authors concluded that assessment of the ω-3 status of study participants, both at baseline and throughout the study, is critical to implementing an effective nutritional intervention. A recently published large scale RCT showing positive results with EPA supplementation met many of the suggestions put forth by Rice et al. (87): (i) the EPA dose given (4 g/day) was ~4-fold greater than other contemporaneous trials, (ii) the study had an average duration of 4.9 years, (iii) the baseline EPA levels were identical between the placebo and treatment group, and (iv) the plasma EPA content at 1 year was 5-fold higher than at baseline (90). This study, as well as other recent RCTs showing beneficial effects of ω-3 supplementation have been reviewed in detail by O'Keefe et al. (91).

Compared to CVD, there have been very few trials investigating the impact of ω-3 supplementation on lupus outcomes, all of which have very few subjects (*n* < 100) (Table 3). Recent reviews on this subject (5, 92) reveal that approximately half of the clinical trials performed employing ω-3 supplementation in lupus patients report a reduction in disease activity (11–13, 16, 18, 93). Many studies that did not observe a reduction in disease activity reported improvements in other areas, such as a reduction in serum triglycerides (9, 10) or biomarkers of inflammation and oxidative stress (9, 15). A critical impediment to evaluating the efficacy of ω-3 supplementation in these trials is the inconsistency in measuring and reporting the ω-3 levels in subjects. Among lupus studies reporting fatty acid levels, there is variability in units used for reporting [mol% (9, 10), wt% (17, 18, 85), mg/mL (12)], the source [platelets (9, 10, 12, 18), RBCs (11, 85), plasma phospholipids (17, 85)], and the fatty acids reported. To more definitively identify ω-3 levels that are protective against lupus symptoms and flaring requires frequent measurement and consistent reporting of ω-3 status in human patients, in addition to more robust clinical trials.

**Table 3:** Summary of ω-3 HUFA intervention trials in lupus patients.

**Year**	**Author**	**FA dose (EPA+DHA)**	**Supplementation type**	**N**	**Trial duration**	**Measured fatty acids**	**Fatty acids reported**	**Result**
1989	Clark et al. (9)	1.8 g, 5.4 g	MaxEPA fish oil capsules	12	5 wk	Yes—platelets	ARA, EPA, DHA	Decreased Triglycerides and cholesterol
1991	Walton et al. (11)	5.6 g	MaxEPA fish oil capsules	27	12 wk	Yes—RBCs	not reported	Improved disease status
1993	Clark et al. (10)	4.4 g	MaxEPA fish oil capsules	21	1 yr	Yes—Platelets	ARA, EPA, DHA	Improvement, but not in renal function or disease activity
2004	Duffy et al. (12)	0.9 g	MaxEPA fish oil capsules	52	24 wk	Yes—Platelets	EPA, DHA	Improved disease status
2005	Nakamura et al. (17)	1.8 g	EPA, ethyl esters	6	3 mo	Yes—Plasma PL	LA, DGLA, ARA, ALA, EPA, DPA, DHA	Decreased oxidative stress (8-isoprostane)
2008	Wright et al. (18)	3 g	Omacor, EPA/DHA methyl esters	60	24 wk	Yes—Platelets	ARA, EPA, DHA	Decreased SLAM-R, BILAG, FMD, isoprostanes
2013	Bello et al. (14)	3 g	Lovaza, EPA/DHA ethyl esters	85	12 wk	No	-	No change
2015	Arriens et al. (13)	4.5g	Metagenics fish oil capsules	32	6 mo	No	-	Improved disease status
2015	Lozovoy et al. (16)	300 mg	Fish oil capsules (no brand)	62	4 mo	No	-	Decreased SLEDAI, increased adiponectin, decreased leptin
2017	Borges et al. (15)	1.28 g	Naturalis HiOmega3 fish oil capsules	49	12 wk	No	-	Decreased CRP

*ARA, arachidonic acid; EPA, eicosapentaenoic acid; DHA, docosahexaenoic acid; LA, linoleic acid; DGLA, dihomo-gamma linoleic acid; ALA, alpha linolenic acid; DPA, docosapentaenoic acid; SLAM-R, systemic lupus activity measure—revised; BILAG, British Isles lupus assessment group; FMD, flow mediated dilation; SLEDAI, systemic lupus erythematosus disease activity index; CRP, C-reactive protein; PL, phospholipid*.

The recent studies identifying the protective effects of ω-3 supplementation in CVD support the potential benefit for a similar dietary intervention in lupus. Notably, patients with lupus have an increased risk of myocardial infarction and CVD mortality relative to the general population (94). A key mechanism proposed to link these chronic diseases is increased oxidative stress (95, 96). A 2012 clinical trial with >700 participants reported that 4 g/day IPE (iscosapent ethyl, an ethyl ester of EPA) for 12 weeks significantly decreased plasma oxLDL (97), an oxidized biomarker implicated in CVD. Similarly, urinary F2 isoprostanes, produced by the non-enzymatic oxidation of arachidonic acid and a widely accepted marker for oxidative stress, were decreased by supplementation with 4 g/day of either DHA or EPA in a study of 59 hypertensive patients with type 2 diabetes (98) A specific member of the F2-isoprostane family, 8-isoprostane, was found to be decreased in with ω-3 supplementation in lupus patients, as measured in both the platelets and urine (Table 3) (17, 18).

In the present study, O3Is above 10% and ω-3 HUFA scores >40% appeared to be associated with absence of disease progression. This is consistent with studies showing decreased mortality from cardiovascular disease in populations where the ω-3 HUFA score is >40% (99) and associating increased ω-3 HUFA scores to a reduction in chronic pain (100). In 2004, Harris and von Schacky proposed that an O3I > 8% was associated with decreased risk of death from CHD, while O3I < 4% was associated with increased risk (101), based on a small clinical trial of 57 subjects. In 2017, a meta-analysis of 10 cohort studies, with a combined *n* > 27,505, confirmed these cutoffs (102). Because there are far fewer clinical studies investigating the role of ω-3 HUFAs in rheumatic disease, and even fewer that present enough fatty acid information to calculate the ω-3 HUFA score, it is difficult to identify a protective ω-3 HUFA score or O3I for lupus. However, a study performed in patients with rheumatoid arthritis showed that increasing the ω-3 HUFA score from ~30 to ~40% resulted in decreased joint swelling, pain, various inflammatory markers, and NSAID and glucocorticoid use (103).

Providing sufficient levels of ω-3 supplementation is paramount to achieving ω-3 HUFA levels capable of reducing symptoms involved in lupus flares. A recent study presented an equation to predict the change in the O3I using the baseline O3I and the supplemented dose of EPA and DHA (104). These findings suggested that individuals with a baseline O3I around 4%, a level typical for many individuals consuming a Western diet, would require 1,500 mg/day EPA + DHA for 13 weeks to achieve an O3I of 8%. Though many ω-3 supplementation trials in lupus patients use doses >1,500 mg/day, the results presented herein suggest that a human equivalent dose of ~5 g/day may be necessary to provide protection against a variety of lupus associated endpoints. Consumption of 5 g/day ω-3 HUFAs has been determined as safe by the European Food Safety Authority after analyzing the impact of ω-3 HUFAs on endpoints such as bleeding time, immune function, and changes in blood LDL-cholesterol (105).

A potential limitation of this study is the limited range of doses of DHA provided (0, 2, and 5 g/day human equivalent dose), which may contribute to the relatively low *R*^2^ value observed between the ω-3 biomarkers and some inflammatory endpoints. Additional intermediate doses of DHA, and corresponding intermediate ω-3 levels, may allow for a more accurate regression model. Other informative modifications to the diet would include using EPA as the primary source of dietary ω-3 HUFAs, providing ω-3 HUFAs as phospholipids rather than triglycerides, or varying levels of ω-6 fatty acids to determine the extent to which ω-6 HUFAs impact levels of ω-3 biomarkers and lupus-associated inflammatory endpoints. Finally, it should be recognized that DHA was administered here prophylactically. Since the most severe lupus symptoms are episodic and associated with flaring, the study design of our experiments is most relevant to periods of disease remission achievable by treatment with glucocorticoids, antimalarials, and immunosuppressants—drugs that have many adverse side effects (106). ω-3 supplementation might be amenable as a substitute or adjunct therapy for these strong drugs to prevent flaring and prolong the quiescent state. However, the prophylaxis model does not mimic the human situation where ω-3 supplementation is provided after the onset of overt symptoms. Thus, further research is needed on the effects of ω-3 supplementation to treat ongoing lupus flares.

To summarize, we demonstrated with this study that both ω-3 HUFA scores and O3Is of mice fed a wide range of diets supplemented with DHA could be related to numerous lupus-associated inflammatory endpoints. This determination is highly relevant to current and future trials investigating the effect of ω-3 supplementation in inflammatory and autoimmune diseases. Our results suggest that measurement of RBC ω-3 levels allows clinicians and administrators of randomized clinical trials to assess the efficacy of the supplementation strategy employed, as well as confirming compliance. Precision nutritional interventions can be designed to reduce consumption of ω-6 fatty acids while simultaneously supplementing with ω-3 HUFAs, with the objective of achieving an ω-3 HUFA score or O3I that may protect against lupus flaring and autoimmune disease progression.

## Data Availability Statement

All datasets presented in this study are included in the article/Supplementary Material.

## Ethics Statement

The animal study was reviewed and approved by The Institutional Animal Care and Use Committee at Michigan State University (AUF#01/15-021-00; AUF# PROTO201800113).

## Author Contributions

KW: investigation, data curation, data analysis/interpretation, figure preparation, manuscript preparation, and project funding. RS, AB, LR, JH: data analysis/interpretation and manuscript preparation. AL: tissue, red blood cells, and diet fatty acid analysis. JP: initial study design, manuscript preparation, supervision, and project funding. All authors contributed to the article and approved the submitted version.

## Conflict of Interest

The authors declare that the research was conducted in the absence of any commercial or financial relationships that could be construed as a potential conflict of interest.

## Supplementary Material

Supplementary Data Sheet 1 Supplementary Figures S1 and S2.

Supplementary Table 1 Microsoft Excel table of RBC fatty acid composition.

Supplementary Table 2 Excel tables containing data from studies 1–3 used to perform generate graphs presented herein.
