# Supplementary material for: Requisite Omega-3 HUFA Biomarker Thresholds for Preventing Murine Lupus Flaring
Source: Front Immunol. 2020 Aug 21;11:1796. doi: 10.3389/fimmu.2020.01796 (PMC7473030; doi:10.3389/fimmu.2020.01796)
Supplement: Supplementary Data Sheet 1 — Supplementary Figures S1 and S2. [file Data_Sheet_1.DOCX]

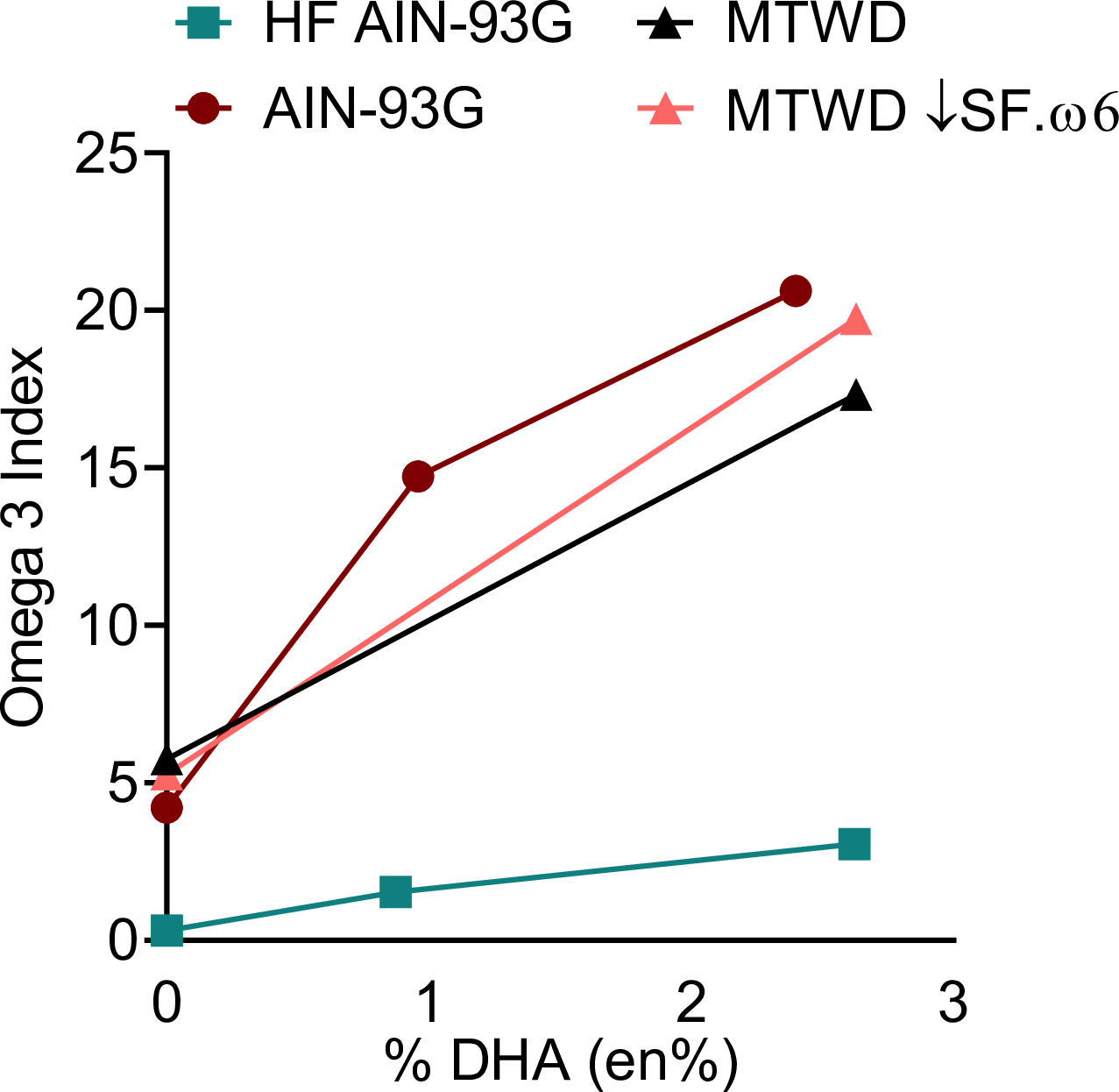


**Figure S1. O3I increases with DHA intake in NZBWF1 mice.** Animals were supplemented with DHA on the background of distinct diets, as described in **Figure 1**. RBCs were collected at experiment termination for fatty acid analysis by GLC (full fatty acid profiles presented in **Table S1**). The DHA content in the diet is presented as en% and the ω-3 content of the RBCs is expressed as the O3I. While animals in Studies 2 and 3 (fed the AIN-93G and MTWD diets) had similar O3Is, animals from Study 1 (fed the HF-AIN-93G diet) had significantly lower O3Is. The analysis of the RBC fatty acids for Study 1 were performed in a different lab from those of Studies 2 and 3, which may account for the observed difference.


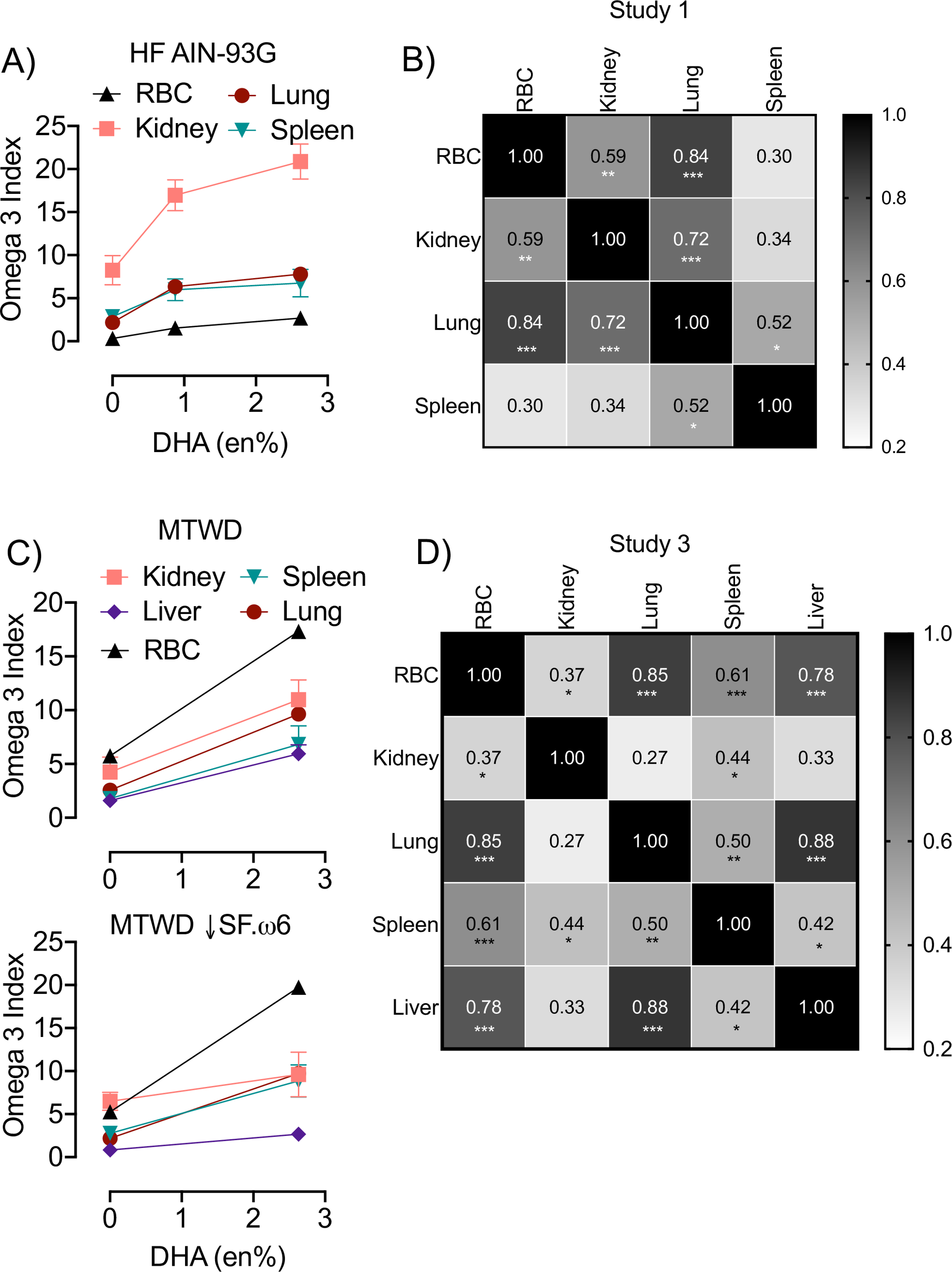


**Figure S2. RBC O3Is do not closely reflect tissue EPA+DHA levels**. Animals from Study 1 (HF AIN-93G (**A, B**)) and Study 3 (MTWD and MTWD SF.ω6 diet (**C, D**)) were analyzed separately to assess the impact of DHA supplementation on RBC and tissue fatty acid incorporation **(A,C).** The O3I was clearly distinct among different tissues when animals were supplemented with DHA. **(B,D)** Spearman’s correlation was used to identify correlations between the RBC O3I and O3I across multiple tissues (**p*<0.05, ***p*<0.01, ****p*<0.001)
